# Machine learning associated with respiratory oscillometry: a computer-aided diagnosis system for the detection of respiratory abnormalities in systemic sclerosis

**DOI:** 10.1186/s12938-021-00865-9

**Published:** 2021-03-25

**Authors:** Domingos S. M. Andrade, Luigi Maciel Ribeiro, Agnaldo J. Lopes, Jorge L. M. Amaral, Pedro L. Melo

**Affiliations:** 1 Electronic Engineering Post-Graduation Program, State University of Rio de Janeiro, Rio de Janeiro, Brazil; 2 Pulmonary Function Laboratory, State University of Rio de Janeiro, Rio de Janeiro, Brazil; 3 Department of Electronics and Telecommunications Engineering, Rio de Janeiro State University, Rio de Janeiro, Brazil; 4 Biomedical Instrumentation Laboratory, Institute of Biology Roberto Alcantara Gomes and Laboratory of Clinical and Experimental Research in Vascular Biology (BioVasc), State University of Rio de Janeiro – Haroldo Lisboa da Cunha Pavilion, number 104 and 105, São Francisco Xavier Street 524 Maracanã, Rio de Janeiro, RJ Zip Code: 20.550-013 Brazil

**Keywords:** System identification techniques, Clinical decision support system, Machine learning, Forced oscillation technique, Diagnostic of respiratory diseases, Systemic sclerosis, Respiratory oscillometry

## Abstract

**Introduction:**

The use of machine learning (ML) methods would improve the diagnosis of respiratory changes in systemic sclerosis (SSc). This paper evaluates the performance of several ML algorithms associated with the respiratory oscillometry analysis to aid in the diagnostic of respiratory changes in SSc. We also find out the best configuration for this task.

**Methods:**

Oscillometric and spirometric exams were performed in 82 individuals, including controls (*n* = 30) and patients with systemic sclerosis with normal (*n* = 22) and abnormal (*n* = 30) spirometry. Multiple instance classifiers and different supervised machine learning techniques were investigated, including k-Nearest Neighbors (KNN), Random Forests (RF), AdaBoost with decision trees (ADAB), and Extreme Gradient Boosting (XGB).

**Results and discussion:**

The first experiment of this study showed that the best oscillometric parameter (BOP) was dynamic compliance, which provided moderate accuracy (AUC = 0.77) in the scenario control group versus patients with sclerosis and normal spirometry (CGvsPSNS). In the scenario control group versus patients with sclerosis and altered spirometry (CGvsPSAS), the BOP obtained high accuracy (AUC = 0.94). In the second experiment, the ML techniques were used. In CGvsPSNS, KNN achieved the best result (AUC = 0.90), significantly improving the accuracy in comparison with the BOP (*p* < 0.01), while in CGvsPSAS, RF obtained the best results (AUC = 0.97), also significantly improving the diagnostic accuracy (*p* < 0.05). In the third, fourth, fifth, and sixth experiments, different feature selection techniques allowed us to spot the best oscillometric parameters. They resulted in a small increase in diagnostic accuracy in CGvsPSNS (respectively, 0.87, 0.86, 0.82, and 0.84), while in the CGvsPSAS, the best classifier's performance remained the same (AUC = 0.97).

**Conclusions:**

Oscillometric principles combined with machine learning algorithms provide a new method for diagnosing respiratory changes in patients with systemic sclerosis. The present study's findings provide evidence that this combination may help in the early diagnosis of respiratory changes in these patients.

**Supplementary Information:**

The online version contains supplementary material available at 10.1186/s12938-021-00865-9.

## Introduction

Systemic sclerosis (SSc) is a chronic connective tissue disease characterized by thickening and fibrosis of the skin and internal organs such as the heart, lungs, kidneys, and gastrointestinal tract [1, 2]. Pulmonary complications are the most common causes of death in SSc, and pulmonary arterial hypertension has become the most crucial life-threatening complication. The most common pulmonary manifestation is interstitial lung disease, associated with pulmonary fibrosis, where the lungs lose their compliance. This abnormality occurs in approximately 80% of cases and is associated with reduced survival [2].

The forced oscillation technique (FOT), also known as respiratory oscillometry, is a system identification method used to evaluate respiratory system resistance and reactance. This method provides a detailed analysis of the respiratory system's mechanical properties, addressing different properties from that evaluated by spirometry, the most traditional method of analyzing respiratory diseases. Indeed, oscillometry is likely complementary to spirometry [3]. The measurement is based on applying low-pressure oscillations to the airway opening to stimulate the respiratory system and measure the associated flow response. Therefore, this technique requires minimal cooperation and no forced expiratory maneuvers and can be used in situations when standard measurements of lung function by spirometry are difficult or not feasible, including children, the elderly, and patients in advanced stages of the disease [4].

Our laboratory successfully applied FOT to obtain a detailed description of the respiratory changes in sarcoidosis [5] and silicosis [6, 7]. Several other research groups also used FOT to diagnose respiratory mechanics changes associated with interstitial lung disease [8–11]. In general, these patients presented increased resistance and frequency-dependency of resistance, as well as more negative reactance. Studies in rheumatoid arthritis showed reactance values and frequency-dependent behavior in resistance significantly different from those of the healthy subjects [12]. Recent studies showed that respiratory reactance reflects fibrosis and restrictive ventilatory deficiency in idiopathic pulmonary fibrosis [13]. FOT allows a simple, not invasive, and detailed analysis of the respiratory system [4]. Taken together, these previous results and features indicate that FOT is a promising tool to facilitate the diagnosis of respiratory abnormalities in patients with SSc.

However, oscillometry is not currently widely used in pulmonary function tests, even with these important clinical advantages. This limitation arises because this method is based on concepts derived from the electrical engineering area, which are not easily interpreted in the clinical environment. Thus, although oscillometry exams are simple, the interpretation of resistance and reactance curves and the derived parameters is difficult for the busy untrained pulmonologist, requiring training and experience.

Machine learning (ML) algorithms have been offered an important contribution to improving lung function tests [4]. In the particular case of oscillometry, previous studies provided clear evidence that these algorithms simplify the interpretation of the results, and therefore, the clinical use of oscillometry [14, 15]. There is also evidence that these algorithms’ use may help increase diagnostic accuracy [14, 16]. Despite the high potential of combining these two methods in lung diseases, there are no previous studies using oscillometry combined with ML methods to diagnose respiratory changes in SSc.

In this context, we hypothesized that using ML methods associated with respiratory oscillometry analysis would improve the diagnosis of respiratory changes in systemic sclerosis. This paper has two key aims. First, to evaluate several ML algorithms to aid in the diagnostic of respiratory changes in SSc. Second, to find out the best configuration for this task.

The next four sections of this paper initially provided a description of the patient groups and the measurement protocol in “Materials and methods” section. We also describe the investigated classifiers, the indexes used for performance evaluation, and the experimental design. The findings of the research are presented in the third section. These findings are discussed and criticized in the fourth section. Finally, the “Conclusion” section summarizes this research’s primary outcomes, focusing on the two key proposed objectives.

## Results

The biometric and spirometric features of the studied subjects are exhibited in Table 1. The three studied groups’ biometric features were similar, and there were no significant differences between the groups. As shown in Table 1, patients with SSc presented significant reductions in the spirometric parameters (*p* < 0.05).

**Table 1:** Anthropometric spirometric characteristics of the studied subjects [mean ± SD and (minimal–maximal values)]

	Control (1) (*n* = 30)	Normal to the exam (2) (*n* = 22)	Altered to the exam (3) (n = 30)	
Age (years)	49.7 ± 13.5 (27–78)	49.6 ± 14.5 (15–78)	46.1 ± 12.8 (21–68)	ns
Body mass (kg)	59.2 ± 68.7 (43.6–77.0)	60.8 ± 11.9 (34.6–88.4)	59.9 ± 13.1 (36.0–88.4)	ns
Height (m)	1.6 ± 5.3 (1.5–1.7)	1.6 ± 3.4 (1.5–1.7)	1.6 ± 6.3 (1.5–1.8)	ns
BMI (kg/m^2^)	23.5 ± 2.9 (18.7–28.6)	24.8 ± 3.9 (16.2–32.5)	24.1 ± 4.3 (16.0–32.8)	ns
Male/female	1/29	1/21	1/29	–
FVC (L)	3.4 ± 0.7	2.8 ± 0.7	2.0 ± 0.5	1–2–3–1
FVC (%)	111.9 ± 18.5	96.7 ± 12.5	64.3 ± 11.6	1–2–3–1
FEV_1_ (L)	2.8 ± 0.6	2.4 ± 0.6	1.7 ± 0.5	1–2–3–1
FEV_1_ (%)	112.5 ± 18.2	97.1 ± 11.6	66.4 ± 12.1	1–2–3–1
FEV_1_/FVC	92.5 ± 10.4	83.4 ± 4.0	86.6 ± 5.5	1–2.3–1
FEF_25–75%_ (L)	3.6 ± 1.0	3.0 ± 0.9	2.5 ± 0.9	1.2–3–1
FEF_25–75%_ (%)	117.4 ± 37.4	112.9 ± 27.2	88.6 ± 28.4	1.2–3–1
FEF/FVC	98.6 ± 28.1	110.0 ± 23.4	134.4 ± 47.8	1.2.3–1

The last column describes the comparisons between groups, in which the dot means non-significant change, while the dash means significant change

FVC: forced vital capacity; FEV_1_: forced expiratory volume in the first second; FEF_25–75%_: forced expiratory flow between 25 and 75%; Ns: not significant; %: percentile of the predicted values

**Fig. 1:**
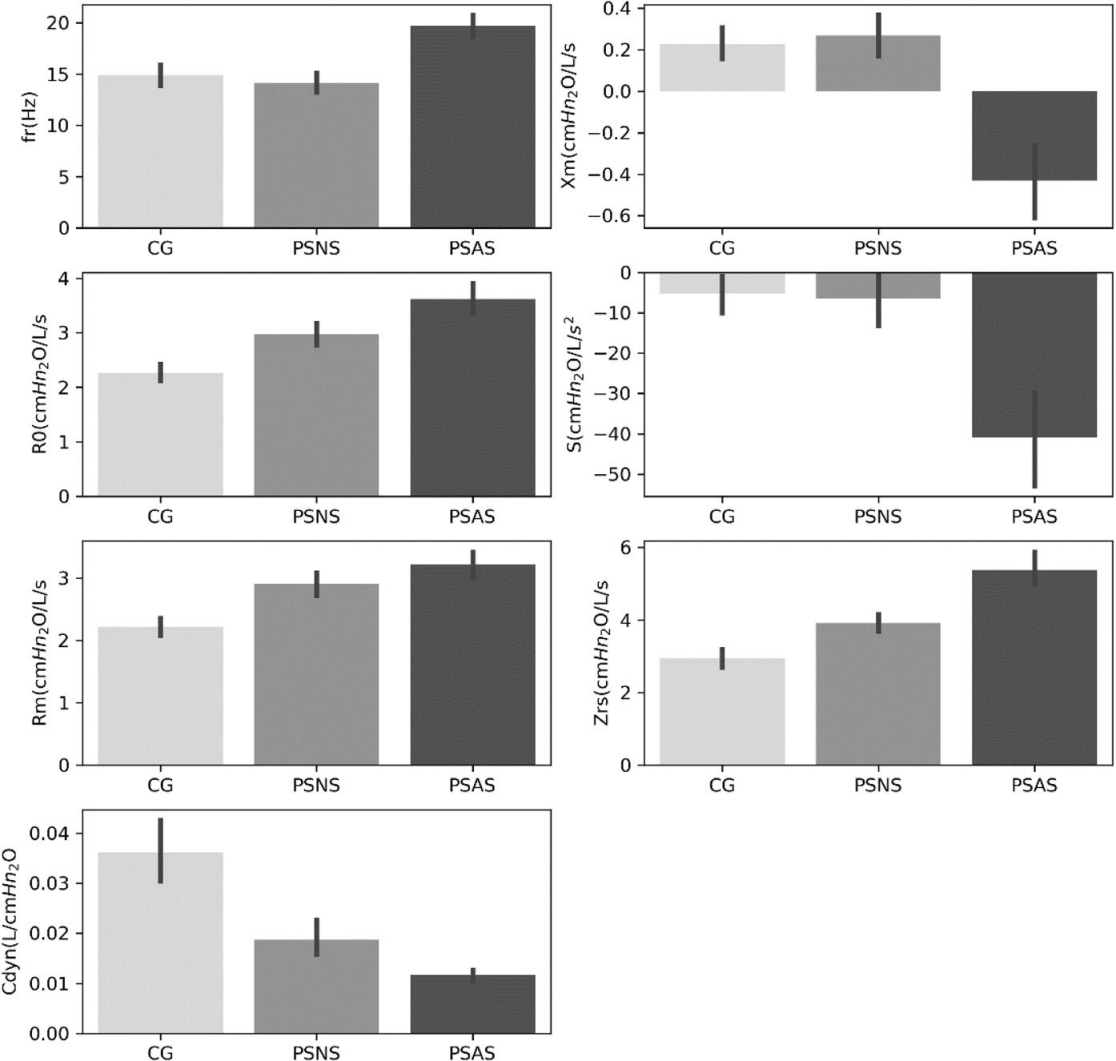
Mean values ± 95% confidence interval of each FOT parameter. Control group (CG), patients with sclerosis and normal spirometry (PSNS), and patients with sclerosis and altered spirometry (PSAS). The analysis of variance (ANOVA) showed that all parameters presented a significant difference in their respective mean values (*p* < 0.001)

The bar charts in Fig. 1 show the characteristics of individuals from the control group (CG), patients with sclerosis and normal spirometry (PSNS), and patients with sclerosis and altered spirometry (PSAS). The mean values of each oscillometric parameter were calculated at a 95% confidence interval. Using the analysis of variance (ANOVA), all oscillometric parameters showed a significant difference in their respective mean values (*p* < 0.001). An increase was observed in the mean values of *R*_0_, *R*_m_, and Zrs in patients with Systemic Sclerosis. Thus, disease carriers have higher resistance values (*R*_0_, *R*_m_) and higher impedance values (Zrs). On the other hand, resonance frequency (fr) and the slope of the resistance curve (*S*) have close CG and PSNS group values. However, fr has higher values for PSAS, and more negative values for *S*. Cdyn has higher values for the CG and similar values in the PSNS and PSAS groups.

**Fig. 2:**
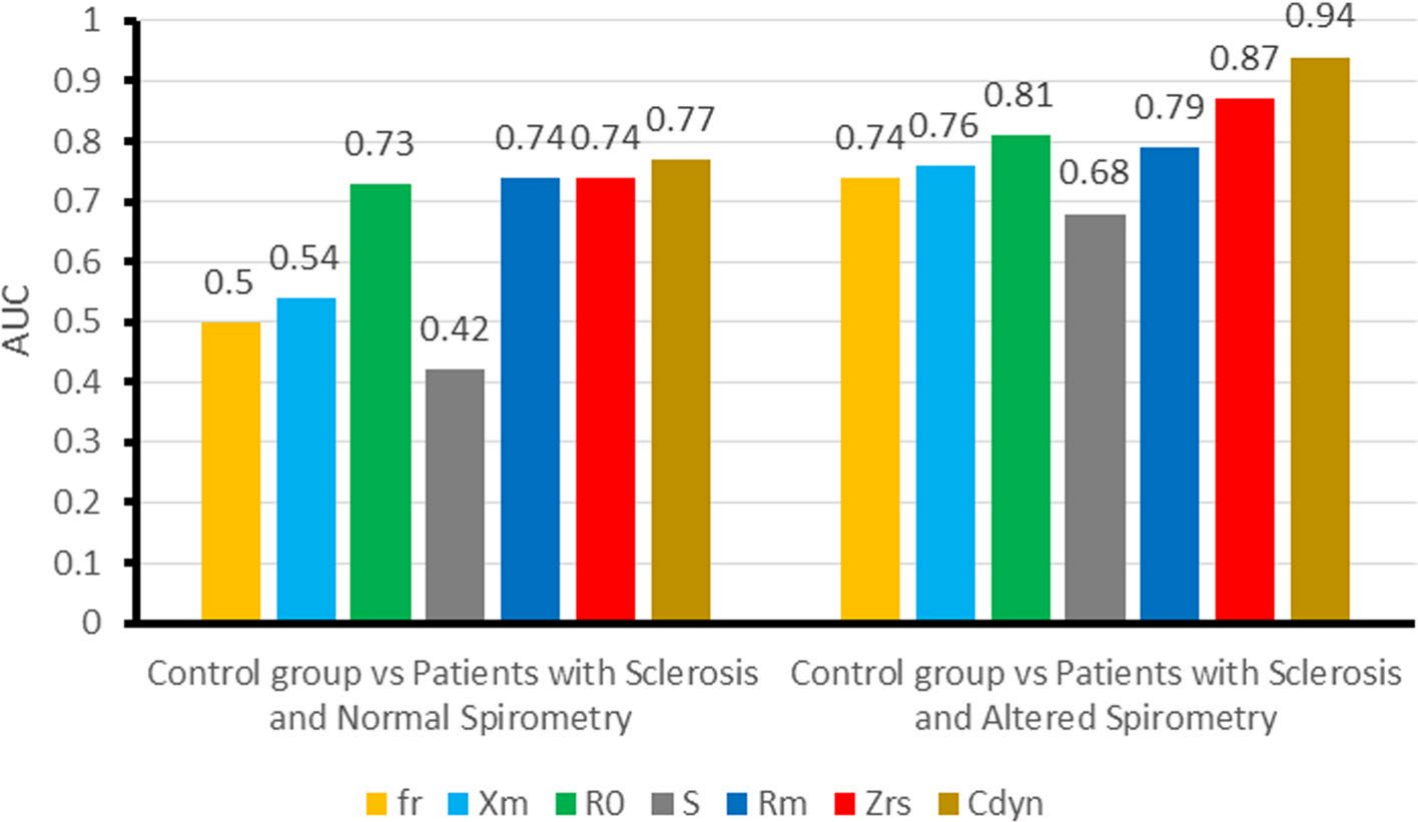
Results of experiment 1, describing the diagnostic accuracy of oscillometry in sclerosis. fr: resonance frequency; *X*_m_: mean respiratory reactance; *R*_0_: respiratory resistance extrapolated at 0 Hz; *S*: slope of the linear relationship of resistance versus frequency; *R*_m_: mean respiratory resistance; Zrs: absolute value of respiratory impedance in 4 Hz; Cdyn: respiratory system dynamic compliance

Figure 2 resumes the results of experiment 1. One can see that Cdyn is the best oscillometric parameter (BOP) to discriminate SSc, presenting moderate diagnostic accuracy (AUC = 0.77) for the situation CGvsPSNS and presenting high diagnostic accuracy (AUC = 0.94) in the scenario CGvsPSAS. Tables and figures with more detail about these results can be found in the supplement (Additional file 1: Tables S1, S2, Additional file 2: Figures S1, and S2).

**Fig. 3:**
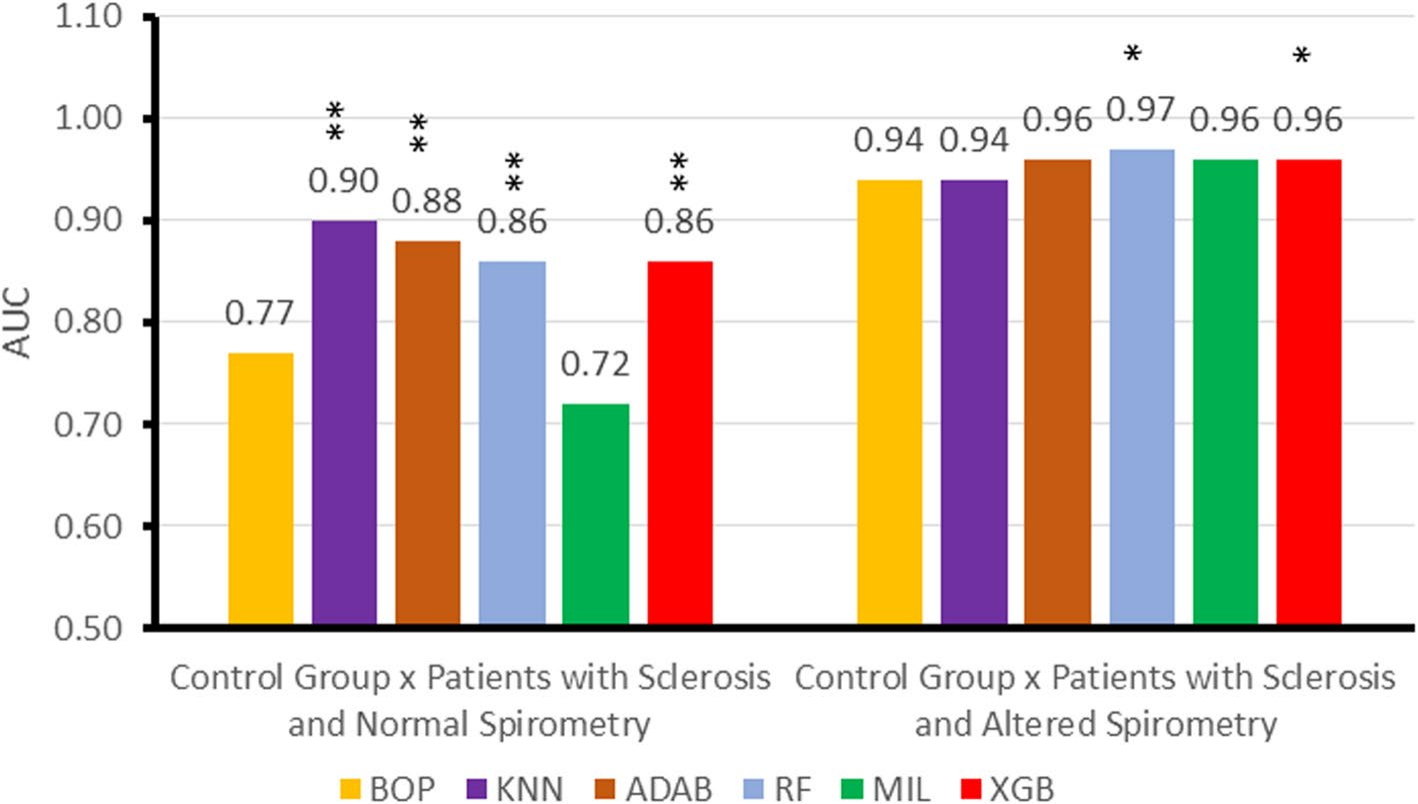
Comparative analysis of the diagnostic accuracy in experiment 2, considering the best oscillometric parameter (BOP) obtained without the use of classifiers), machine learning algorithms, and the MIL classifier. *K-NN* K-Nearest Neighbor, *ADAB* Adaboost with decision tree classifiers, *RF* Random Forests, *MIL* Multiple Instance Learning, *XGB* Extreme Boosting Gradient Classifiers, *AUC* area under the ROC curve. Also, “*” indicates that there a statistically significant difference comparing to BOP (*p* < 0.05) and “**” (*p* < 0.01)

Figure 3 presents the AUCs of the BOP, the ML algorithms, and the MIL classifier obtained in experiment 2. One can see that the ML algorithms improved the AUC in the situation CGvsPSNS. KNN achieved the best result with AUC = 0.90. This result indicates that the algorithm provides a highly accurate diagnosis (0.9 ≤ AUC ≤ 1.0). The second-best performance was realized by the ADAB, with AUC = 0.88. The AUCs’ comparison with the methodology proposed by Delong et al. [17] has shown that KNN, ADAB, RF, and XGB presented a statistically significant difference concerning the BOP.

In the scenario, CGvsPSAS, ADAB, RF, and XGB could provide a small improvement in the AUC. RF and XGB exhibited a statistically significant difference regarding the BOP, while the RF classifier has achieved the best performance with AUC = 0.97. Table 2 presents the five oscillometric parameters selected in these experiments.

**Table 2:** Five oscillometric parameters selected by MIL and RFE

	Control group versus patients with sclerosis and normal spirometry (CGvsPSNS)	Control group versus patients with sclerosis and altered spirometry (CGvsPSAS)
MIL	Xm, R0, S, Rm, Cdin	fr, R0, Rm, Zrs, Cdyn
RFE	fr, R0, Rm, Zrs, Cdin	Fr, Xm, Rm, Zrs, Cdin

fr: resonance frequency; *X*_m_: mean respiratory reactance; *R*_0_: respiratory resistance extrapolated at 0 Hz; *S*: slope of the linear relationship of resistance versus frequency; *R*_m_: mean respiratory resistance; Zrs: absolute value of respiratory impedance in 4 Hz; Cdyn: respiratory system dynamic compliance

**Fig. 4:**
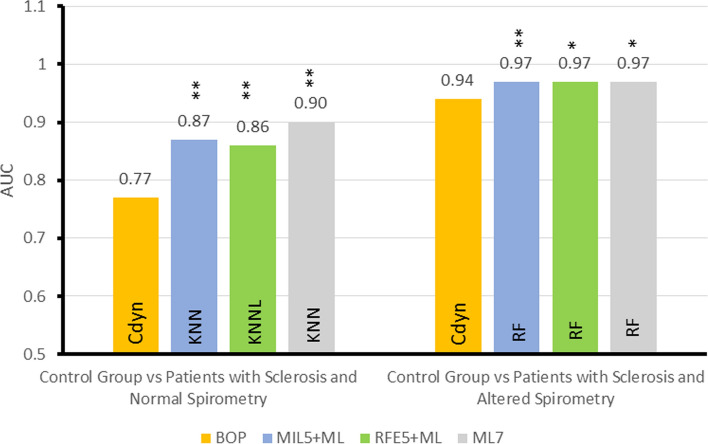
Summary of Experiment 3 (MIL5 + ML: MIL as five feature selector) and Experiment 4 (RFE5 + ML: RFE as a five feature selector)—AUCs for the best oscillometric parameter (BOP), for the best ML algorithms in experiments 3 and 4, and the best ML algorithm with oscillometric parameters (ML7). The figure indicates the best oscillometric parameter and the best ML algorithm in each case. Also, “*” indicates that there a statistically significant difference comparing to BOP (*p* < 0.05) and “**” (*p* < 0.01)

Figure 4 shows the results of experiment 3, presenting the AUCs obtained by the following strategies: BOP, best ML algorithms with all seven oscillometric parameters (ML7), best ML algorithm with five oscillometric parameters selected by MIL (MIL5 + ML), and the best ML algorithm with five oscillometric parameters selected by RFE (RFE5 + ML).

Regarding MIL’s selection, when one compares obtained AUCs with those obtained in the second experiment, it is worth noting that there is only a small decrease in the scenario CGvsPSNS. In the CGvsPSAS scenario, the obtained AUCs stay the same. In both situations, the AUCs’ comparison with the methodology proposed by Delong et al. has shown a statistically significant difference concerning the BOP. KNN achieves the best AUC in the scenario CGvsPSNS with feature selection done by MIL (AUC = 0.87), while in the CGvsPSAS, the best AUC was obtained by RF (AUC = 0.97).

**Fig. 5:**
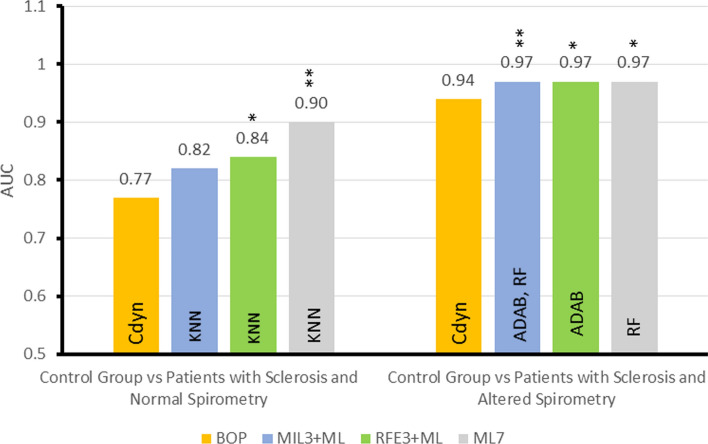
Summary of Experiment 5 (MIL3 + ML: MIL as three feature selector) and Experiment 6 (RFE3 + ML: RFE as a three feature selector)—AUCs for the best oscillometric parameter (BOP), for the best ML algorithms in experiments 5 and 6, and the best ML algorithm with oscillometric parameters (ML7). The figure indicates the best oscillometric parameter and the best ML algorithm in each case. Also, “*” indicates that there a statistically significant difference comparing to BOP (*p* < 0.05) and “**” (*p* < 0.01)

The fifth and sixth experiments were designed to train ML algorithms with the selection of the three best features. Table 3 presents the selected features, and Fig. 5 resumes the results.

**Table 3:** Three oscillometric parameters selected by MIL and RFE

	Control group versus patients with sclerosis and normal spirometry (CGvsPSNS)	Control group versus patients with sclerosis and altered spirometry (CGvsPSAS)
MIL	*S*, *R*_m_, Cdyn	*R*_0_, Zrs, Cdyn
RFE	*R*_0_, Zrs, Cdyn	*R*_m_, Zrs, Cdyn

*R*_0_: respiratory resistance extrapolated at 0 Hz; *S*: slope of the linear relationship of resistance versus frequency; *R*_m_: mean respiratory resistance; Zrs: absolute value of respiratory impedance in 4 Hz; Cdyn: respiratory system dynamic compliance

**Fig. 6:**
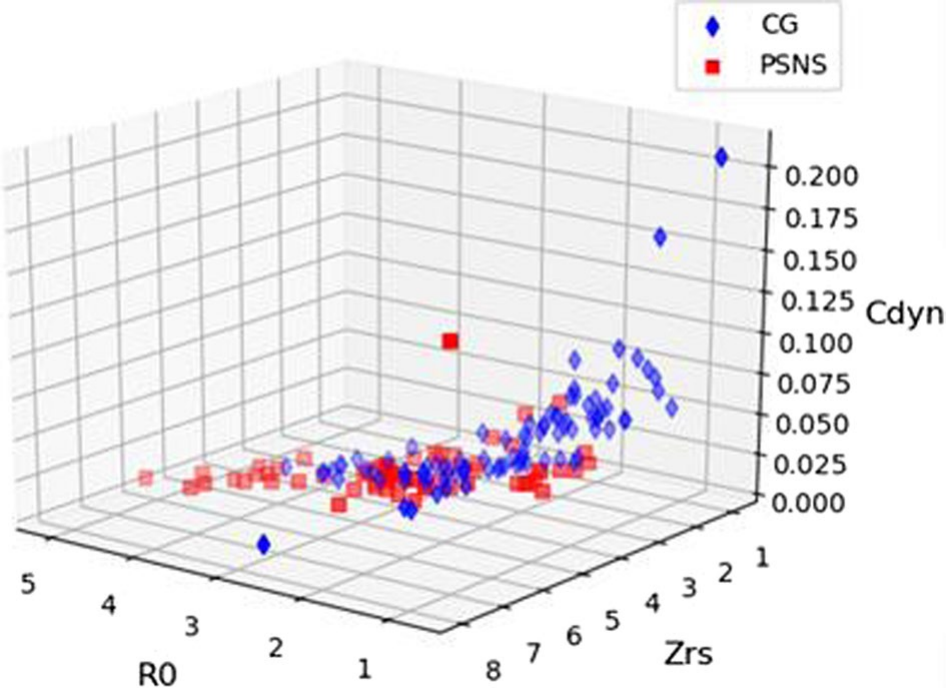
Representation of the dataset CGvsPSNS using three features: *R*_0_, Zrs, and Cdyn

Even with only three features, AUCs’ comparison has shown a statistically significant difference concerning the BOP in both cases. In CGvsPSNS, once again, there is a small decrease in the performance (AUC = 0.84). In the CGvsPSAS, the performance was the same (AUC = 0.97). Figure 6 presents a 3D picture of the CG and PSNS.

**Fig. 7:**
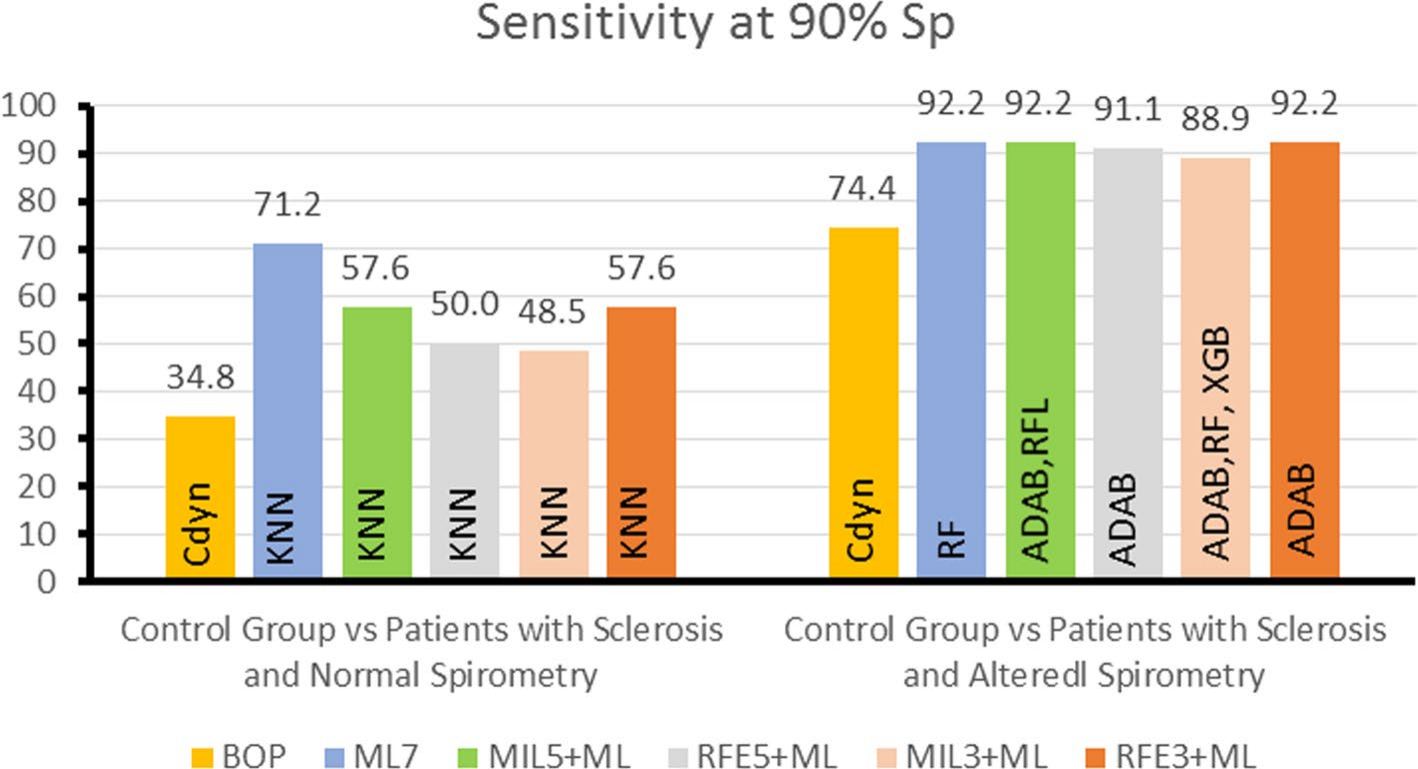
Summary of the experiments describing comparisons of the sensitivity at 90% Sp obtained using the best oscillometric parameter (BOP) and ML methods in all experiments. The sensitivity at 90% Sp presented is that of the best classifier

**Fig. 8:**
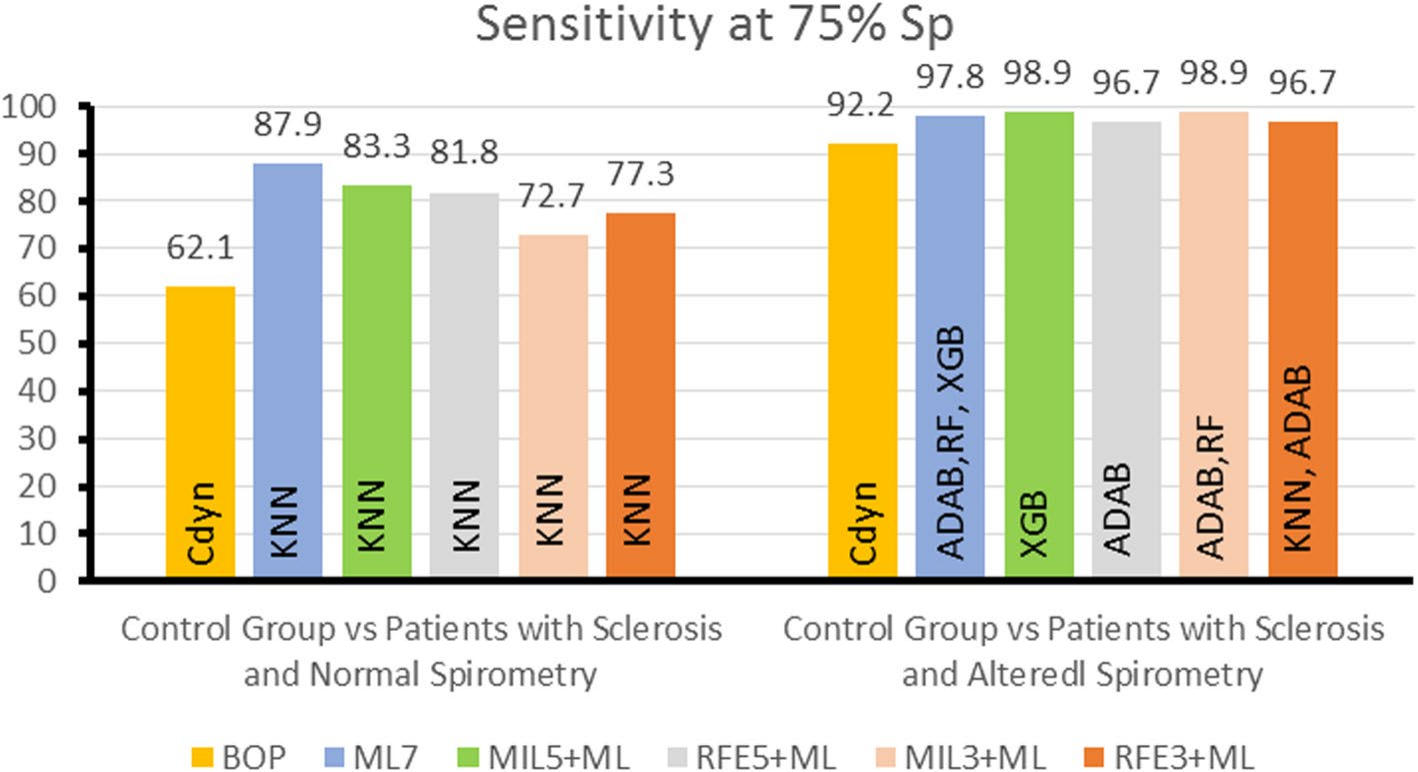
Summary of the experiments describing comparisons of the sensitivity at 75% Sp obtained using the best oscillometric parameter (BOP) and ML methods in all experiments. The sensitivity at 75% Sp presented is that of the best classifier

For additional analysis of the ROC curves, Figs. 7 and 8 show, respectively, the Se observed at an Sp of 90% and at an Sp of 75% (representing bearable specificity). We included the 90% specificity level since it allows only 10% false positives, introducing the most difficult cases into the correct group. It is also noticeable that the sensitivities at 90% Sp of the best ML classifiers were higher than those observed using the BOP in all performed experiments. Best ML classifiers invariably presented better results than BOP at 75% Sp.

The interested reader may find a detailed description of the results obtained in experiments 2 to 6 in the supplement (Additional file 1: Tables S3 to S22, Additional file 2: Figures S3 to S22).

## Discussion

This is the first study on designing an automatic classifier to assist in diagnosing respiratory abnormalities in patients with SSc using respiratory oscillometry. It was shown that it could simplify lung function’s clinical evaluation and improve these exams’ diagnostic accuracy.

In the first experiment, the dynamic compliance (Cdyn) was the oscillometric parameter that obtained the best individual performance in both scenarios: CGvsPSNS and CGvsPSAS. These findings are in close agreement with the diffuse fibrosis that affects the interstitium and alveolar septa in SSc [1, 2]. They are also in line with the reduction in compliance observed by Greenwald et al. [18] using the esophageal balloon technique and the increase in reactance area obtained by Aronsson et al. [19] using impulse oscillometry.

In the first scenario, as expected, due to the small differences in the measured parameters (Fig. 1), it was challenging to separate the control group from the patients with sclerosis and normal spirometry, which yields an AUC = 0.77, indicating moderate diagnostic accuracy (Fig. 2). In the second scenario, the increase in physiological abnormalities resulted in increased differences in the measured parameters (Fig. 1). This allowed Cdyn to easily separate the two groups and present an AUC = 0.94, which stands for high diagnostic accuracy (Fig. 2). These results are consistent with previous studies showing increased Cdyn diagnostic accuracy with physiological abnormalities in sarcoidosis [5], adults with sickle cell anemia [20], and COPD [21].

In the second experiment (Fig. 3), it is possible to note that the best result for the scenario CGvsPSNS was achieved by the KNN (AUC = 0.90). The use of the KNN, ADAB, RF, and XGB algorithms resulted in a significant improvement in diagnostic accuracy. KNN was followed by ADAB, RF, and XGB using all oscillometric parameters, with ADAB remarkably adjacent to high diagnostic accuracy (AUC = 0.88). In accordance with the present results, previous studies have demonstrated similar increase in accuracy in sickle cell anemia [22], the differential diagnosis of asthma and restrictive respiratory diseases [23], and the early diagnosis of smoking-induced respiratory changes [16].

Similar to previous studies [23, 24], feature selection allowed the reduction of the used features without a significant reduction in performance (Fig. 4). In CGvsPSNS, feature selection helped spot the most relevant features. Although the methods selected a different set of features, there is a significant intersection (*R*_0_, *R*_m_, and Cdyn), which agrees with what can be seen in Fig. 1. In the other scenario, CGvsPSAS, RF’s best results were achieved, followed by XGB and ADAB (Fig. 4). For this scenario, the feature selection has shown that the same results could be achieved using fewer oscillometric parameters. As mentioned in the Introduction, pulmonary manifestation in SSc is characterized by interstitial lung disease associated with pulmonary fibrosis [2]. In this sense, one interesting finding was obtaining Cdyn between the most relevant features in the two studied scenarios (Table 2). This is in close accordance with pathophysiological fundamentals involved in this disease, in which the lungs lose their compliance [2].

Figure 5 summarizes all the results obtained in the fifth and sixth experiments and compares the results in experiments 1 and 2. In CGvsPSNS, the feature selection did not increase diagnostic accuracy, but it indicated important features *R*_0_, Zrs, and Cdyn, which agrees with Fig. 1. The use of the reduction of attributes was intended to reduce the complexity of the analysis. The current study found selected features consistent with the presence of lung fibrosis [1, 2]. This rather interesting result is consistent with clinically relevant abnormalities that are known to be associated with reduced survival in these patients [2]. The three selected main features allowed us to visually inspect the division between groups. These results further support the idea of a simple visual analysis to help the clinical use of FOT [23]. This optimized interpretation allowed us to observe that the SSc presents smaller values for Cdyn and higher values for R0 and Zrs. Due to its direct physiological translation, this simple spatial description may help interpret the proposed medical decision support system’s results, contributing to its use in the clinical scenario.

Concerning the use of the MIL algorithm, it was efficient selecting attributes (Tables 2 and 3), where it was able to achieve a better result for the control versus normal spirometric analysis than the one obtained by the specialist selection. However, the MIL algorithm was not as efficient as the traditional classifiers (Figs. 3, 4, 5, 7, and 8).

Recent studies have shown the importance of improving our respiratory system knowledge [25] and the non-invasive lung function tests [26–28]. Respiratory oscillometry has been widely perceived as the state-of-the-art lung function analysis [29], and one of the most promising emerging technologies in this area [3, 30]. However, although its advantages associated with a detailed and straightforward examination are particularly important, this method is not yet widely used. One of the main aspects limiting its wide routine application is that the obtained indexes' interpretation is based on electric models, requiring training and practice. Previous research has established that diagnostic easiness is a fundamental attribute for occupied non-specialist clinicians [31]. The present study supports previous evidence [14, 16] and contributes to this direction showing that ML algorithms can improve SSc patients’ medical services, simplifying the use of respiratory oscillometry and improving the diagnosis of the cited disease.

Early diagnosis of the abnormal respiratory changes in SSc could support early intervention, thus possibly restricting the disease’s progression, mitigating adverse symptoms, improving general well-being, restraining complications and comorbidities, and early mortality. Artificial intelligence/machine learning methods have improved pulmonary function analysis since the 1980s [4]. The current study extends these findings providing evidence that a combination of respiratory oscillometry and a clinical decision support system based on ML techniques might indicate early abnormal respiratory changes in SSc.

Finally, some important limitations need to be considered and clarified to the reader. First, the study was limited to the Brazilian population at a specific practice site. Thus, it is not possible to ensure its generalizability to a different population. It is suggested that multicenter data be investigated in future studies to expand the generalizability of the findings. It is worth mentioning that by examining the adopted inclusion and exclusion criteria and the present study’s biometric features, readers can easily evaluate whether they are likely to achieve similar findings in their patient population. It is also pertinent to mention that the experimental design of the present work enhances its generalizability. Globally recognized inclusion and exclusion criteria were used, and the work was conducted under usual clinical procedures in a typical setting.

Second, SSc is a disease of low incidence, making it hard to obtain a high number of patients. As a result, the datasets available are relatively small, which requires care to control the complexity of the ML models. In addition to all the care taken in this study to avoid overfitting, such as controlling the hyperparameters, feature selection can also help control overfitting by diminishing the inputs. Another reason to employ feature selection is that a smaller number of features can help simplify the analysis. Moreover, if one uses only three features, it is possible to visualize the separation between groups, which can aid the diagnostic explanation.

## Conclusion

We designed and tested various classifier methods to achieve a clinical decision support system to assist in detecting respiratory abnormalities in patients with systemic sclerosis. The respiratory oscillometry parameters alone can only reach moderate diagnostic accuracy (AUC = 0.77) in scenario CGvsPSNS. The ML classifiers' use allowed us to enhance accuracy, reaching high accuracy (AUC ≥ 0.9) in this situation, representing the disease’s initial stages. In the CGvsPSAS, the oscillometric parameter alone could reach high diagnostic accuracy (AUC = 0.94); nevertheless, ML algorithms could provide a small enhancement (AUC = 0.97). The developed system may also help simplify oscillometry use in detecting respiratory changes in patients with systemic sclerosis. Notably, the adoption of feature selection has spotted the most crucial oscillometric parameters, which simplify the analysis. Taken together, the results of the present study and these practical considerations provide clear evidence that respiratory oscillometry combined with machine learning classifiers’ may help to improve lung function tests in systemic sclerosis.

This study’s next steps include improving the understanding and management of systemic sclerosis by integrating ML algorithms and telemedicine systems based on respiratory oscillometry.

## Materials and methods

Eighty-two volunteers were included in the study. Fifty-two presented SSc, and 30 were healthy, composing the control group. The patients with SSc were divided into two groups: (1) the normal spirometry group (*n* = 22), which included patients diagnosed with SSc and showing normal spirometry, and (2) the altered spirometry group (*n* = 30) that was composed of patients diagnosed with SSc and presenting altered spirometry, associated with restrictive ventilatory disorder [2].

The exams were conducted at the Pulmonary Function Testing Laboratory of the Pedro Ernesto University Hospital and the Biomedical Instrumentation Laboratory of the State University of Rio de Janeiro. The Hospital Ethical Committee approved the study (approval number 456 CEP/HUPE), and all subjects gave informed written consent. This study is in agreement with The Declaration of Helsinki. The inclusion criteria in the present study were a confirmed diagnosis of SSc according to the American College of Rheumatology [32], including volunteers from both genders. The exclusion criteria were a history of exacerbation of disease in the previous 90 days, smoking, and presence of tuberculosis or pneumonia, chronic lung diseases, presence of respiratory infections in the previous 30 days, chest trauma or surgery, inability to perform the tests, and chemotherapy and radiotherapy for cancer.

The control group was composed of healthy volunteers from both genders without a history of cardiovascular or lung disease or smoking. These individuals did not present respiratory infections and showed normal spirometry [33].

**Fig. 9:**
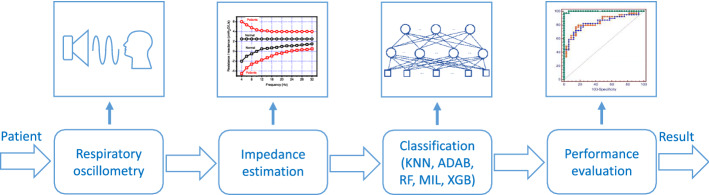
Simplified block diagram describing the main steps in this study. *K-NN* K-Nearest Neighbor, *ADAB* Adaboost with decision tree classifiers, *RF* Random Forests, *MIL* Multiple Instance Learning, *XGB* Extreme Boosting Gradient Classifiers

The main elements in this study are the respiratory oscillometry measurements, impedance estimation, and clinical decision support system development and performance evaluation. The complete process is shown in Fig. 9. Each operation will be described in the next sections.

### Respiratory oscillometry measurements and parameters

These analyses were performed using as input excitation small amplitude pressure oscillations (≤ 2 cmH_2_O), which were produced by a loudspeaker and applied during tidal breathing at the entrance of the individual’s airway through the oral cavity. The result of the exams was generated as the mean of three tests, each 16 s long. These tests were considered adequate if they were free of pauses and presented stable rate and tidal volumes. A pseudo-random noise signal between 4 and 32 Hz was used, and the exams are repeated until all analyzed frequencies presented the minimal coherence function of 0.9. We used a coefficient of variability ≤ 10% in the lowest frequency (4 Hz) in the three used tests to avoid outlying values. The experiments were conducted using an impedance analyzer described previously [34].

Linear regression in the respiratory resistance values in the 4–16 Hz range was used to interpret the obtained results. This yielded resistance at 0 Hz (*R*_0_), the mean resistance in this frequency range (*R*_m_), and the slope of the relationship between the resistive values and frequency (*S*). *R*_0_ describes the low-frequency range. This parameter integrates the Newtonian effects related to the airways, lung, and chest wall resistance and the effect of gas redistribution [35]. The mid-frequency range is described by *R*_m_, which reflects the resistance in the central airways [36]. *S* is associated with ventilation non-homogeneities [37].

The reactive results were interpreted using four indexes: the mean reactance (*X*_m_), resonance frequency (fr), the impedance module (Zrs), and the dynamic compliance (Cdyn). *X*_m_ was calculated using the 4- to 32-Hz frequency range and describes ventilation inhomogeneity. The fr occurs when the elastic and inertive properties cancel out, and the respiratory reactance becomes zero [38]. Cdyn was calculated based on the reactance at 4 Hz (Cdyn = 1/2*π*fX4) and reflects the respiratory compliance, comprising pulmonary, chest wall, and airway compliances. This parameter is also associated with ventilation homogeneity [36]. Zrs includes the effects of resistance and elastic loads in 4 Hz, representing the respiratory system’s total mechanical load [39].

### Data sets

In the present work, experiments were executed in a dataset that consisted of 246 measurements acquired from the volunteers. Healthy volunteers contributed with 90 measurements of the oscillometric parameters, patients with sclerosis and normal spirometry with 66, and patients with sclerosis and altered spirometry supplied 90 measurements.

### Machine learning algorithms

Machine learning algorithms can discover crucial relationships among the features in a data set [4, 40]. These models’ inference can be carried out with minimal user intervention through several techniques such as linear models, graphic models, ensemble strategies, hybrid approaches, and artificial neural networks, among others. In our previous research [14, 15, 24], we have experimented with a wide diversity of models and concluded that ensemble strategies had outstanding performance. In this study, we want to investigate the Extreme Gradient Boosting (XGB) algorithm, a type of ensemble derived from gradient boosting. The final inference model is an assemblage of weak inference models, routinely decision trees. It builds the model in a stepwise mode, where its step is designed to model the error of the previous ones. XGB implements Gradient Boosting, focusing on regularization to control overfitting, which gives it better performance. Besides, we also want to explore Multiple Instance Learning (MIL) to the early examination of respiratory changes in patients with systemic sclerosis. Therefore, in this study, the following ML algorithms were appraised:

- K-Nearest Neighbor (KNN) [41];
- Adaboost with decision trees [42];
- Random Forest (RF) [43];
- Extreme Gradient Boosting (XGB) [44];
- Multiple Instance Learning (MIL) [45];

The first three algorithms have already been briefly described in the previous studies [14, 15, 24]; therefore, we will provide a condensed description of the two algorithms that have not been used in our studies before. A complete description of them can be found in the references.

The Extreme Gradient Boosting is a more efficient, regularized version of Gradient Boosting. In Gradient Boosting, one fits an additive model (ensemble) in a forward manner. There is an introduction of a weak learner to cope with the previous weak learners’ shortcomings in each stage. These shortcomings can be described by the residuals (errors) left by the previous weak learners. Hence, the weak learner to be added must fit the residuals to the ensemble to produce better results. The relation of this algorithm with gradient descent (GD) is since the residuals can be seen as negative gradients, and the GD can employ them to locate the minimum value of the loss function. Common choices for the loss function are root mean squared error (regression) and log-loss (classification).

The multi-instance learning (MIL) paradigm was introduced by [45] focused on an application in biochemistry. MIL is considered an extension of supervised learning, where the labels are assigned to a set of instances, known as bags, and not to each instance individually. MIL’s central idea is related to the notion of bags: it is labeled as a negative bag (Bi−) if the total instances contained in it are negative and labeled as positive (Bi+) if, at best, one of the instances is positive. In this way, a bag can be defined as a collection of instances or regions. The Diverse Density (DD) algorithm was originally introduced by [46], where the algorithm is described as an assessment of the intersection of positive bags minus the union of negative bags. The algorithm’s central idea is to find a concept point in the feature space close to at least one instance of each positive bag and far from the negative bag instances.

### Experimental design

This study executed a total of six experiments. The purpose of the first experiment was to investigate the proficiency of a single oscillometric parameter alone to correctly spot the airway obstruction level in patients with systemic sclerosis. We considered two different situations: the control group versus patients with sclerosis and normal spirometry (CGvsPSNS) and the control group versus patients with sclerosis and altered spirometry (CGvsPSAS). The remaining experiments also evaluate the two situations described.

The second experiment exploited ML algorithms and compared them with the results obtained by a single oscillometric parameter to reveal if the ML algorithms could achieve superior performance. The area under the ROC curve (AUC) was then chosen as the measurement of the performance since it is regularly employed in medicine [47–50] and yields a superior way to confront classifiers than accuracy [51]. We did not implement feature selection; thus, all of the oscillometric indexes were used. The classifiers described previously were realized with Scikit-learn [52], a machine learning library written in python. On the other hand, Multiple Instance Learning was implemented by the library described in [53]. Since the dataset contains only 246 oscillometric measurements, the k-fold validation procedure [54] is indicated to allow the valuation of the generalization proficiency in the whole dataset. Hyperparameter tuning is a crucial step in model selection. Scikit-learn possesses several strategies to allow hyperparameter fine-tuning, such as grid search, which experiments with all possible combinations of the hyperparameters. Table J0 presents the classifiers and their respective chosen hyperparameters for tuning.

The third experiment evaluates the capability of MIL as a feature selector with the purpose of complexity reduction and to gain knowledge about the importance of different oscillometric parameters [55]. Its role is to select five oscillometric parameters in a previous step before the classifier training. The fourth experiment employs the recursive feature selection (RFE) to select five oscillometric parameters before the classifier training. RFE is a wrapper strategy that can use several ML algorithms to assess the performance. In this paper, the ML algorithm’s choice was the linear support vector machine classifier with L1 regularization. The fifth experiment uses MIL to select three oscillometric parameters, and the sixth employs RFE to choose three oscillometric parameters.

The hypothesis test is a requisite for contrasting ML algorithms. There are a wide variety of parametric tests available, which are commonly based on the *t*-test [40, 56, 57]. Some of the nonparametric tests most used are McNemar’s and Wilcoxon’s [56, 58, 59]. In this work, the hypothesis test was carried out with AUCs by applying the methodology specified in Delong et al. [17].

## Supplementary Information

**Additional file 1.** **Table S1** Results of the experiment 1 (Control group versus Patients with sclerosis and normal spirometry). The Area Under the ROC Curve (AUC), the Standard Error (SE) and the 95% confidence interval (95% CI) of each FOT parameter. **Table S2** Results of the experiment 1 (Control group x Patients with sclerosis and altered spirometry). The Area Under the ROC Curve (AUC), the Standard Error (SE) and the 95% confidence interval (95% CI) of each FOT parameter.

**Additional file 2.** **Figure S1** ROC curves for Experiment 1. ROC curves for each one of the FOT parameters - Control Group versus Patients with Sclerosis and Normal Spirometry (CGvsPSNS). **Figure S2** ROC curves for Experiment 1. ROC curves for each one of the FOT parameters - Control Group versus Patients with Sclerosis and Altered Spirometry (CGvsPSAS).

## Data Availability

The datasets used or analyzed during the current study are available from the corresponding author on reasonable request.

## Abbreviations

%: Percentile of the predicted values
ADAB: AdaBoost with decision trees
ANOVA: Analysis of variance
AUC: Area under the receiver operating characteristic curve
BOP: Best oscillometric parameter
Cdyn: Dynamic compliance
CG: Control group
DD: Diverse density algorithm
FOT: Forced oscillation technique
FEV_1_: Forced expiratory volume in the first second
fr: Resonant frequency
FVC: Forced vital capacity
FEF_25–75%_: Forced expiratory flow between 25 and 75%
KNN: K-Nearest Neighbors
MIL: Multiple instance learning
MIL5 + ML: Best ML algorithm with 5 oscillometric parameters selected by MIL
ML: Machine learning
ML7: Best ML algorithms with all 7 oscillometric parameters
Ns: Not significant
PSNS: Patients with sclerosis and normal spirometry
PSAS: Patients with sclerosis and altered spirometry
RF: Random Forest
RFE: Recursive feature selection
RFE5 + ML: Best ML algorithm with 5 oscillometric parameters selected by RFE
*R*_0_: Intercept resistance
*R*_m_: Mean resistance in the 4–16 Hz range
ROC: Receiver operating characteristic curve
*S*: Angular coefficient of resistance in the 4–16 Hz range
Se: Sensitivity
Sp: Specificity
SSc: Systemic sclerosis
XGB: Extreme Gradient Boosting algorithm
*X*_4_: Respiratory reactance at 4 Hz
Zrs: Absolute value of respiratory impedance in 4 Hz
*X*_m_: Mean reactance evaluated considering the 4 to 32 Hz frequency range
