# Supplementary material for: Machine learning associated with respiratory oscillometry: a computer-aided diagnosis system for the detection of respiratory abnormalities in systemic sclerosis
Source: Biomed Eng Online. 2021 Mar 25;20:31. doi: 10.1186/s12938-021-00865-9 (PMC7995797; doi:10.1186/s12938-021-00865-9)
Supplement: Supplementary file 1 — Additional file 1. Table S1 Results of the experiment 1 (Control group versus Patients with sclerosis and normal spirometry). The Area Under the ROC Curve (AUC), the Standard Error (SE) and the 95% confidence interval (95% CI) of each FOT parameter. Table S2 Results of the experiment 1 (Control group x Patients with sclerosis and altered spirometry). The Area Under the ROC Curve (AUC), the Standard Error (SE) and the 95% confidence interval (95% CI) of each FOT parameter. [file 12938_2021_865_MOESM1_ESM.docx]

Table S1: Results of the experiment 1 (Control group versus Patients with sclerosis and normal spirometry). The Area Under the ROC Curve (AUC), the Standard Error (SE) and the 95% confidence interval (95% CI) of each FOT parameter.

|  | AUC | SE | 95% CI |
| --- | --- | --- | --- |
| fr (Hz) | 0.50 | 0.05 | 0.41-0.59 |
| Xm (cmH_2_O/L/s) | 0.54 | 0.05 | 0.44-0.68 |
| R0 (cmH_2_O/L/s) | 0.73 | 0.04 | 0.65-0.82 |
| S (cmH_2_O/L/s^2^) | 0.42 | 0.05 | 0.33-0.51 |
| Rm (cmH_2_O/L/s) | 0.75 | 0.04 | 0.66-0.83 |
| Zrs4Hz (cmH_2_O/L/s) | 0.74 | 0.04 | 0.66-0.82 |
| Cdyn,rs (L/cmH_2_O) | 0.77 | 0.04 | 0.69-0.84 |

fr: Resonance frequency;

Xm: Mean respiratory reactance;

R0: Respiratory resistance extrapolated at 0 Hz;

S: Slope of the linear relationship of resistance versus frequency;

Rm: Mean respiratory resistance;

Zrs4Hz: Absolute value of respiratory impedance in 4 Hz;

Crs,dyn: Respiratory system dynamic compliance;

Table S2: Results of the experiment 1 (Control group x Patients with sclerosis and altered spirometry). The Area Under the ROC Curve (AUC), the Standard Error (SE) and the 95% confidence interval (95% CI) of each FOT parameter.

|  | AUC | SE | 95% CI |
| --- | --- | --- | --- |
| fr (Hz) | 0.74 | 0.04 | 0.67 – 0.81 |
| Xm (cmH_2_O/L/s) | 0.76 | 0.04 | 0.69 – 0.83 |
| R0 (cmH_2_O/L/s) | 0.81 | 0.03 | 0.75 – 0.88 |
| S (cmH_2_O/L/s^2^) | 0.69 | 0.04 | 0.60 – 0.76 |
| Rm (cmH_2_O/L/s) | 0.79 | 0.03 | 0.72 – 0.86 |
| Zrs4Hz (cmH_2_O/L/s) | 0.87 | 0.03 | 0.82 – 0.92 |
| Cdyn,rs (L/cmH_2_O) | 0.94 | 0.02 | 0.90 – 0.97 |

fr: Resonance frequency;

Xm: Mean respiratory reactance;

R0: Respiratory resistance extrapolated at 0 Hz;

S: Slope of the linear relationship of resistance versus frequency;

Rm: Mean respiratory resistance;

Zrs4Hz: Absolute value of respiratory impedance in 4 Hz;

Crs,dyn: Respiratory system dynamic compliance;

Table S3: Results of the experiment 2 (Control group versus Patients with sclerosis and normal spirometry). Best studied classifiers with the original FOT parameters as inputs. The 95% confidence interval is shown in parenthesis bellow each performance metric. The AUC standard error is also shown in parenthesis.

|  | Se  (%) | Sp  (%) | AUC |
| --- | --- | --- | --- |
| BFP | 62.1  (50.4 - 73.8) | 77.7  (69.2 - 86.4) | 0.77(0.04)  (0.69 - 0.84) |
| KNN | 86.4  (78.1 - 94.6) | 79.0  (70.4-87.3) | 0.90(0.03)  (0.85-0.95) |
| ADAB | 81.8  (74.3 – 92.3) | 80.0  (72.5-91.1) | 0.88(0.03)  (0.82-0.94) |
| RF | 77.3  (67.2 – 87.4) | 78.9  (70.4 - 87.3) | (0.86(0.03)  (0.80 – 0.92) |
| XGB | 87.9  (80.0 – 95.7) | 71.1  (62.0 – 80.0) | 0.86(0.03)  (0.79 – 0.92) |
| MIL | 83.3  (74.3 – 92.3) | 57.7  (47.6 – 68.0) | 0.72(0.04)  (0.64 - 0.81) |

BFP: Best FOT parameter (obtained without the use of classifiers)

KNN: K Nearest Neighbors

ADAB: Adaboost with decision tree classifiers;

RF: Random Forests;

XGB: XGBoost;

MIL: Multiple Instance Learning;

Table S4: Comparison of AUCs- Experiment 2 (Control group versus Patients with sclerosis and normal spirometry).

|  | KNN | ADAB | RF | XGB | MIL |
| --- | --- | --- | --- | --- | --- |
| BFP | 0.134±0.039** | 0.111±0.033** | 0.092±0.028** | 0.089±0.032** | 0.043±0.043* |
| KNN | - | 0.023±0.022 | 0.042±0.024 | 0.045±0.027 | 0.177±0.043** |
| ADAB | - | - | 0.018±0.013 | 0.022±0.015 | 0.154±0.037** |
| RF | - | - | - | 0.004±0.015 | 0.135±0.035** |
| XGB |  |  |  |  | 0.132±0.037** |

BFP: Best FOT parameter (obtained without the use of classifiers)

KNN: K Nearest Neighbors

ADAB: Adaboost with decision tree classifiers;

RF: Random Forests;

XGB: XGBoost;

MIL: Multiple Instance Learning;

* p<0.05

** p<0.01

Table S5: Results of the experiment 2 (Control group x Patients with sclerosis and altered spirometry). Best studied classifiers with the original FOT parameters as inputs. The 95% confidence interval is shown in parenthesis bellow each performance metric. The AUC standard error is also shown in parenthesis.

|  | Se  (%) | Sp  (%) | AUC |
| --- | --- | --- | --- |
| BFP | 87.8  (81.0 – 94.5) | 85.6  (78.3 – 92.8) | 0.94(0.02)  (0.90-0.97) |
| KNN | 86.7  (79.6 - 93.7) | 87.8  (81.0 – 94.5 | 0.94(0.02)  (0.90 – 0.97) |
| ADAB | 88.9  (82.4 – 95.4) | 93.3  (88.2 – 98.5) | 0.96(0.02)  (0.93 – 0.99) |
| RF | 92.2  (86.7 – 97.7) | 91.1  (85.2 – 96.9) | 0.97(0.01)  (0.94 – 0.99) |
| XGB | 91.1  (85.2 – 97.0) | 91.1  (85.2-97.0) | 0.96(0.01)  (0.94 – 0.99) |
| MIL | 90.0  (83.8 – 96.2) | 85.5  (78.3 – 92.8) | 0.94(0.02)  (0.90-0.97) |

BFP: Best FOT parameter (obtained without the use of classifiers)

KNN: K Nearest Neighbors

ADAB: Adaboost with decision tree classifiers;

RF: Random Forests;

XGB: XGBoost;

MIL: Multiple Instance Learning;

Table S6: Comparison of AUCs- Experiment 2 (Control group x Patients with sclerosis and altered spirometry).

|  | KNN | ADAB | RF | XGB | MIL |
| --- | --- | --- | --- | --- | --- |
| BFP | 0.003±0.020 | 0.027±0.015 | 0.032±0.013* | 0.029±0.014* | 0.002±0.008 |
| KNN | - | 0.024±0.012* | 0.029±0.013* | 0.026±0.017 | 0.000±0.018 |
| ADAB | - | - | 0.005±0.007 | 0.002±0.011 | 0.025±0.015 |
| RF | - |  |  | 0.003±0.007 | 0.029±0.013 |
| XGB |  |  |  |  | 0.026±0.015 |

BFP: Best FOT parameter (obtained without the use of classifiers)

KNN: K Nearest Neighbors

ADAB: Adaboost with decision tree classifiers;

RF: Random Forests;

XGB: XGBoost;

MIL: Multiple Instance Learning;

* p<0.05

** p<0.01

Table S7: Results of the experiment 3 (Control group versus Patients with sclerosis and normal spirometry). Best studied classifiers with 5 FOT parameters selected by MIL as inputs. The 95% confidence interval is shown in parenthesis bellow each performance metric. The AUC standard error is also shown in parenthesis.

|  | Se  (%) | Sp  (%) | AUC |
| --- | --- | --- | --- |
| BFP | 62.1  (50.4 - 73.8) | 77.7  (69.2 - 86.4) | 0.77(0.04)  (0.69 - 0.84) |
| KNN | 81.8  (72.5-91.1) | 78.9  (70.4 – 87.3) | 0.87(0.03)  (0.81 – 0.93) |
| ADAB | 83.3  (74.3-92.3) | 74.4  (65.4-83.4) | 0.86(0.03)  (0.80 – 0.92) |
| RF | 74.2  (63.7 – 84.8) | 82.2  (74.3 – 90.1) | 0.84(0.03)  (0.77-0.91) |
| XGB | 86.4  (78.1 – 94.6) | 70.0  (60.5 – 79.5) | 0.84(0.03)  (0.78- 0.91) |
| MIL | 71.2  (60.3 – 82.1) | 67.7  (58.1 – 77.4) | 0.74(0.04)  (0.66 – 0.82) |

BFP: Best FOT parameter (obtained without the use of classifiers)

KNN: K Nearest Neighbors

ADAB: Adaboost with decision tree classifiers;

RF: Random Forests;

XGB: XGBoost;

MIL: Multiple Instance Learning;

Table S8: Comparison of AUCs- Experiment 3 (Control group versus Patients with sclerosis and normal spirometry).

|  | KNN | ADAB | RF | XGB | MIL |
| --- | --- | --- | --- | --- | --- |
| BFP | 0.101±0.034** | 0.094±0.034** | 0.074±0.030* | 0.077±0.034* | 0.026±0.022 |
| KNN | - | 0.007±0.018 | 0.027±0.018 | 0.024±0.023 | 0.127±0.37** |
| ADAB | - | - | 0.020±0.014 | 0.017±0.017 | 0.120±0.036** |
| RF | - | - | - | 0.003±0.016 | 0.100±0.033** |
| XGB |  |  |  |  | 0.103±0.034** |

BFP: Best FOT parameter (obtained without the use of classifiers)

KNN: K Nearest Neighbors

ADAB: Adaboost with decision tree classifiers;

RF: Random Forests;

XGB: XGBoost;

MIL: Multiple Instance Learning;

* p<0.05

** p<0.01

Table S9: Results of the experiment 3 (Control group versus Patients with sclerosis and altered spirometry). Best studied classifiers with 5 FOT parameters selected by MIL as inputs. The 95% confidence interval is shown in parenthesis bellow each performance metric. The AUC standard error is also shown in parenthesis.

|  | Se  (%) | Sp  (%) | AUC |
| --- | --- | --- | --- |
| BFP | 87.8  (81.0 – 94.5) | 85.6  (78.3 – 92.8) | 0.94(0.02)  (0.90-0.97) |
| KNN | 91.1  (85.2 – 97.0) | 88.9  (82.4-95.4) | 0.95(0.02)  (0.92 – 0.98) |
| ADAB | 92.2  (86.7 – 97.7) | 93.3  (88.2- 98.5) | 0.97(0.01)  (0.94 – 0.99) |
| RF | 93.3  (88.2 – 98.4) | 90.0  (83.8 – 96.2) | 0.97(0.01)  (0.95-1.00) |
| XGB | 93.3  (88.2 – 98.4) | 90.0  (83.8 – 96.2) | 0.96(0.01)  (0.93 – 0.99) |
| MIL | 90.0  (83.8 – 96.2) | 85.5  (78.3 – 92.8) | 0.94(0.02)  (0.90 – 0.97) |

ADAB: Adaboost with decision tree classifiers;

KNN: K Nearest Neighbors

RF: Random Forests

LGB: Light Gradient Boost

XGB: Extreme Gradient Boost

MIL: Multiple Instance Learning

Table S10: Comparison of AUCs- Experiment 3 (Control group versus Patients with sclerosis and altered spirometry).

|  | KNN | ADAB | RF | XGB | MIL |
| --- | --- | --- | --- | --- | --- |
| BFP | 0.013±0.017 | 0.029±0.017 | 0.036±0.013** | 0.025±0.016 | 0.003±0.008 |
| KNN | - | 0.016±0.013 | 0.023±0.011* | 0.012±0.011 | 0.011±0.015 |
| ADAB | - | - | 0.007±0.009 | 0.004±0.015 | 0.027±0.017 |
| RF | - | - | - | 0.011±0.011 | 0.034±0.014* |
| XGB |  |  |  |  | 0.023±0.0172 |

BFP: Best FOT parameter (obtained without the use of classifiers)

KNN: K Nearest Neighbors

ADAB: Adaboost with decision tree classifiers;

RF: Random Forests;

XGB: XGBoost;

MIL: Multiple Instance Learning;

* p<0.05

** p<0.01

Table S11: Results of the experiment 4 (Control group versus Patients with sclerosis and normal spirometry). Best studied classifiers with 5 FOT parameters selected by Recursive Feature Selection. The 95% confidence interval is shown in parenthesis bellow each performance metric. The AUC standard error is also shown in parenthesis.

|  | Se  (%) | Sp  (%) | AUC |
| --- | --- | --- | --- |
| BFP | 62.1  (50.4 - 73.8) | 77.7  (69.2 - 86.4) | 0.77(0.04)  (0.69 - 0.84) |
| KNN | 80.3  (70.7 – 89.9) | 77.7  (69.2 – 86.4) | 0.86(0.03)  (0.80 – 0.92) |
| ADAB | 80.3  (70.7 – 89.9) | 78.9  (70.4 – 87.3) | 0.83(0.03)  (0.77 – 0.90) |
| RF | 80.3  (70.7 – 89.9) | 74.4  (65.4 – 83.4) | 0.85(0.03)  (0.79 – 0.91) |
| XGB | 71.2  (60.3 – 82.1) | 78.9  (70.4 – 87.3) | 0.84(0.03)  (0.78 – 0.91) |
| MIL | 75.8  (65.4 – 86.1) | 62.2  (52.2 – 72.2) | 0.73(0.04)  (0.65 – 0.82) |

BFP: Best FOT parameter (obtained without the use of classifiers)

KNN: K Nearest Neighbors

ADAB: Adaboost with decision tree classifiers;

RF: Random Forests;

XGB: XGBoost;

MIL: Multiple Instance Learning;

Table S12: Comparison of AUCs- Experiment 4 (Control group versus Patients with sclerosis and normal spirometry).

|  | KNN | ADAB | RF | XGB | MIL |
| --- | --- | --- | --- | --- | --- |
| BFP | 0.092±0.035** | 0.067±0.036 | 0.084±0.031** | 0.075±0.032* | 0.030±0.02 |
| KNN | - | 0.025±0.020 | 0.007±0.019 | 0.017±0.02 | 0.122±0.038** |
| ADAB | - | - | 0.017±0.012 | 0.008±0.017 | 0.098±0.040* |
| RF | - | - | - | 0.009±0.011 | 0.114±0.036* |
| XGB |  |  |  |  | 0.105±0.037** |

BFP: Best FOT parameter (obtained without the use of classifiers)

KNN: K Nearest Neighbors

ADAB: Adaboost with decision tree classifiers;

RF: Random Forests;

XGB: XGBoost;

MIL: Multiple Instance Learning;

* p<0.05

** p<0.01

Table S13: Results of the experiment 4 (Control group versus Patients with sclerosis and altered spirometry). Best studied classifiers with 5 FOT parameters selected by Recursive Feature Selection. The 95% confidence interval is shown in parenthesis bellow each performance metric. The AUC standard error is also shown in parenthesis.

|  | Se  (%) | Sp  (%) | AUC |
| --- | --- | --- | --- |
| BFP | 87.8  (81.0 – 94.5) | 85.6  (78.3 – 92.8) | 0.94(0.02)  (0.90-0.97) |
| KNN | 83.3  (75.6 – 91.0) | 93.3  (88.2 – 98.5) | 0.94(0.02)  (0.91 - 0.98) |
| ADAB | 90.0  (83.8 – 96.2) | 94.4  (89.7 – 99.2) | 0.97(0.01)  (0.94 – 0.99) |
| RF | 92.2  (86.7 – 97.7) | 88.9  (82.4 – 95.4) | 0.97(0.01)  (0.94 – 0.99) |
| XGB | 88.8  (82.4 – 95.4) | 92.2  (86.7 – 97.8) | 0.96(0.01)  (0.93 – 0.99) |
| MIL | 88.8  (82.4 – 95.4) | 85.5  (78.3 – 92.8) | 0.93(0.02)  (0.89-0.97) |

BFP: Best FOT parameter (obtained without the use of classifiers)

KNN: K Nearest Neighbors

ADAB: Adaboost with decision tree classifiers;

RF: Random Forests;

XGB: XGBoost;

MIL: Multiple Instance Learning;

Table S14: Comparison of AUCs- Experiment 4 (Control group versus Patients with sclerosis and altered spirometry).

|  | KNN | ADAB | RF | XGB | MIL |
| --- | --- | --- | --- | --- | --- |
| BFP | 0.007±0.017 | 0.030±0.014* | 0.032±0.013* | 0.027±0.013* | 0.006±0.005 |
| KNN | - | 0.024±0.010* | 0.025±0.010* | 0.020±0.012 | 0.013±0.018 |
| ADAB | - | - | 0.002±0.005 | 0.004±0.006 | 0.037±0.015* |
| RF | - | - | - | 0.006±0.005 | 0.039±0.015** |
| XGB |  |  |  |  | 0.033±0.014* |

BFP: Best FOT parameter (obtained without the use of classifiers)

KNN: K Nearest Neighbors

ADAB: Adaboost with decision tree classifiers;

RF: Random Forests;

XGB: XGBoost;

MIL: Multiple Instance Learning;

* p<0.05

** p<0.01

Table S15: Results of the experiment 5 (Control group versus Patients with sclerosis and normal spirometry). Best studied classifiers with 3 FOT parameters selected by MIL as inputs. The 95% confidence interval is shown in parenthesis bellow each performance metric. The AUC standard error is also shown in parenthesis.

|  | Se  (%) | Sp  (%) | AUC |
| --- | --- | --- | --- |
| BFP | 62.1  (50.4 - 73.8) | 77.7  (69.2 - 86.4) | 0.77(0.04)  (0.69 - 0.84) |
| KNN | 87.9  (80.0 – 95.7) | 66.7  (60.0 – 76.4) | 0.82(0.03)  (0.75 – 0.89) |
| ADAB | 71.2  (60.3 – 82.1) | 81.1  (73.0 – 89.2) | 0.82(0.04)  (0.75 – 0.89) |
| RF | 84.8  (76.2 – 93.5) | 65.5  (55.7 – 75.4) | 0.80(0.04)  (0.73 – 0.88) |
| XGB | 78.7  (68.9 – 88.6) | 70.0  (60.5 – 79.4) | 0.79(0.04)  (0.71 – 0.86) |
| MIL | 75.8  (65.4 – 86.1) | 66.7  (56.9 – 76.4) | 0.75(0.04)  (0.67 -0.83) |

BFP: Best FOT parameter (obtained without the use of classifiers)

KNN: K Nearest Neighbors

ADAB: Adaboost with decision tree classifiers;

RF: Random Forests;

XGB: XGBoost;

MIL: Multiple Instance Learning;

Table S16: Comparison of AUCs- Experiment 5 (Control group versus Patients with sclerosis and normal spirometry).

|  | KNN | ADAB | RF | XGB | MIL |
| --- | --- | --- | --- | --- | --- |
| BFP | 0.055±0.032 | 0.051±0.033 | 0.037±0.027 | 0.022±0.031 | 0.020±0.022 |
| KNN | - | 0.003±0.024 | 0.018±0.023 | 0.032±0.029 | 0.075±0.034* |
| ADAB | - | - | 0.014±0.016 | 0.029±0.017 | 0.071±0.036* |
| RF | - | - | - | 0.015±0.015 | 0.057±0.030 |
| XGB | - | - | - | - | 0.042±0.034 |

BFP: Best FOT parameter (obtained without the use of classifiers)

KNN: K Nearest Neighbors

ADAB: Adaboost with decision tree classifiers;

RF: Random Forests;

XGB: XGBoost;

MIL: Multiple Instance Learning;

* p<0.05

** p<0.01

Table S17: Results of the experiment 5 (Control group versus Patients with sclerosis and altered spirometry). Best studied classifiers with 3 FOT parameters selected by MIL as inputs. The 95% confidence interval is shown in parenthesis bellow each performance metric. The AUC standard error is also shown in parenthesis.

|  | Se  (%) | Sp  (%) | AUC |
| --- | --- | --- | --- |
| BFP | 87.8  (81.0 – 94.5) | 85.6  (78.3 – 92.8) | 0.94(0.02)  (0.90-0.97) |
| KNN | 94.4  (89.7 – 99.2) | 85.6  (78.3 – 92.8) | 0.96(0.01)  (0.93 – 0.99) |
| ADAB | 94.4  (89.7 – 99.2) | 88.8  (82.4 – 95.4) | 0.97(0.01)  (0.95 – 1.00) |
| RF | 92.2  (86.7 – 97.8) | 88.8  (82.4 – 95.4) | 0.97(0.01)  (0.95 – 1.00) |
| XGB | 91.1  (85.2 – 97.0) | 90.0  (83.8 – 96.2) | 0.96(0.01)  (0.93 – 0.99) |
| MIL | 88.9  (82.4 – 95.4) | 85.6  (78.3 – 92.8) | 0.93  (0.89- 0.97) |

ADAB: Adaboost with decision tree classifiers;

KNN: K Nearest Neighbors

RF: Random Forests

LGB: Light Gradient Boost

XGB: Extreme Gradient Boost

MIL: Multiple Instance Learning

Table S18: Comparison of AUCs- Experiment 5 (Control group versus Patients with sclerosis and altered spirometry).

|  | KNN | ADAB | RF | XGB | MIL |
| --- | --- | --- | --- | --- | --- |
| BFP | 0.021±0.015 | 0.036±0.015* | 0.036±0.013** | 0.025±0.016 | 0.002±0.002 |
| KNN | - | 0.015±0.009 | 0.014±0.009 | 0.003±0.013 | 0.024±0.016 |
| ADAB | - | - | 0.001±0.006 | 0.012±0.012 | 0.039±0.016* |
| RF | - | - | - | 0.011±0.009 | 0.038±0.013** |
| XGB | - | - | - | - | 0.027±0.016 |

BFP: Best FOT parameter (obtained without the use of classifiers)

KNN: K Nearest Neighbors

ADAB: Adaboost with decision tree classifiers;

RF: Random Forests;

XGB: XGBoost;

MIL: Multiple Instance Learning;

* p<0.05

** p<0.01

Table S19: Results of the experiment 6 (Control group versus Patients with sclerosis and normal spirometry). Best studied classifiers with 3 FOT parameters selected by Recursive Feature Selection. The 95% confidence interval is shown in parenthesis bellow each performance metric. The AUC standard error is also shown in parenthesis.

|  | Se  (%) | Sp  (%) | AUC |
| --- | --- | --- | --- |
| BFP | 62.1  (50.4 - 73.8) | 77.7  (69.2 - 86.4) | 0.77(0.04)  (0.69 - 0.84 |
| KNN | 83.3  (74.3 – 92.3) | 71.1  (61.7 – 80.5) | 0.84(0.03)  (0.77 -0.90) |
| ADAB | 74.2  (63.7 – 84.8) | 82.2  (74.3 – 0.90) | 0.83(0.03)  (0.76 -0.90) |
| RF | 71.2  (60.3 – 82.1) | 81.1  (73.0 – 89.2) | 0.83(0.03)  (0.76-0.90) |
| XGB | 71.2  (60.3 – 82.1) | 75.6  (66.7 – 84.4) | 0.81(0.04)  (0.74 – 0.88) |
| MIL | 81.8  (72.5 – 91.1) | 57.8  (47.6 – 67.9) | 0.72(0.04)  (0.64 – 0.80) |

BFP: Best FOT parameter (obtained without the use of classifiers)

KNN: K Nearest Neighbors

ADAB: Adaboost with decision tree classifiers;

RF: Random Forests;

XGB: XGBoost;

MIL: Multiple Instance Learning;

Table S20: Comparison of AUCs- Experiment 6 (Control group versus Patients with sclerosis and normal spirometry).

|  | KNN | ADAB | RF | XGB | MIL |
| --- | --- | --- | --- | --- | --- |
| BFP | 0.072±0.033* | 0.064±0.032* | 0.062±0.027* | 0.043±0.032* | 0.047±0.022* |
| KNN | - | 0.008±0.021 | 0.010±0.020 | 0.029±0.026 | 0.119±0.039** |
| ADAB | - | - | 0.002±0.016 | 0.021±0.020 | 0.111±0.039** |
| RF | - | - | - | 0.019±0.015 | 0.109±0.036** |
| XGB | - | - | - | - | 0.090±0.039 |

BFP: Best FOT parameter (obtained without the use of classifiers)

KNN: K Nearest Neighbors

ADAB: Adaboost with decision tree classifiers;

RF: Random Forests;

XGB: XGBoost;

MIL: Multiple Instance Learning;

* p<0.05

** p<0.01

Table S21: Results of the experiment 6 (Control group versus Patients with sclerosis and altered spirometry). Best studied classifiers with 3 FOT parameters selected by Recursive Feature Selection. The 95% confidence interval is shown in parenthesis bellow each performance metric. The AUC standard error is also shown in parenthesis.

|  | Se  (%) | Sp  (%) | AUC |
| --- | --- | --- | --- |
| BFP | 87.8  (81.0 – 94.5) | 85.6  (78.3 – 92.8) | 0.94(0.02)  (0.90-0.97) |
| KNN | 93.3  (88.2 – 98.5) | 87.8  (81.0 – 94.5) | 0.95(0,02)  (0.92 - 0.98) |
| ADAB | 92.2  (86.7 – 97.7) | 92.2  (86.7- 97.7) | 0.97(0.01)  (0.94 – 0.99) |
| RF | 90.0  (83.8 – 96.2) | 91.1  (85.2 – 97.0) | 0.96(0.01)  (0.93 – 0.99) |
| XGB | 88.9  (82.4 – 95.4) | 93.3  (88.2 – 98.5) | 0.95(0.02)  (0.91 – 0.98) |
| MIL | 87.8  (81.0 – 94.5) | 85.6  (78.3 – 92.8) | 0.93((0.02)  (0.89 – 0.97) |

BFP: Best FOT parameter (obtained without the use of classifiers)

KNN: K Nearest Neighbors

ADAB: Adaboost with decision tree classifiers;

RF: Random Forests;

XGB: XGBoost;

MIL: Multiple Instance Learning;

Table S22: Comparison of AUCs- Experiment 6 (Control group versus Patients with sclerosis and altered spirometry).

|  | KNN | ADAB | RF | XGB | MIL |
| --- | --- | --- | --- | --- | --- |
| BFP | 0.016±0.016 | 0.031±0.016 | 0.025±0.012* | 0.011±0.015 | 0.007±0.005 |
| KNN | - | 0.015±0.011 | 0.010±0.009 | 0.004±0.015 | 0.022±0.017 |
| ADAB | - | - | 0.005±0.007 | 0.020±0.014 | 0.038±0.017 |
| RF | - | - | - | 0.014±0.010 | 0.032±0.014* |
| XGB |  |  |  |  | 0.018±0.016 |

BFP: Best FOT parameter (obtained without the use of classifiers)

KNN: K Nearest Neighbors

ADAB: Adaboost with decision tree classifiers;

RF: Random Forests;

XGB: XGBoost;

MIL: Multiple Instance Learning;

* p<0.05

** p<0.01
