# Supplementary material for: Machine learning associated with respiratory oscillometry: a computer-aided diagnosis system for the detection of respiratory abnormalities in systemic sclerosis
Source: Biomed Eng Online. 2021 Mar 25;20:31. doi: 10.1186/s12938-021-00865-9 (PMC7995797; doi:10.1186/s12938-021-00865-9)
Supplement: Supplementary file 2 — Additional file 2. Figure S1 ROC curves for Experiment 1. ROC curves for each one of the FOT parameters - Control Group versus Patients with Sclerosis and Normal Spirometry (CGvsPSNS). Figure S2 ROC curves for Experiment 1. ROC curves for each one of the FOT parameters - Control Group versus Patients with Sclerosis and Altered Spirometry (CGvsPSAS). [file 12938_2021_865_MOESM2_ESM.pdf]

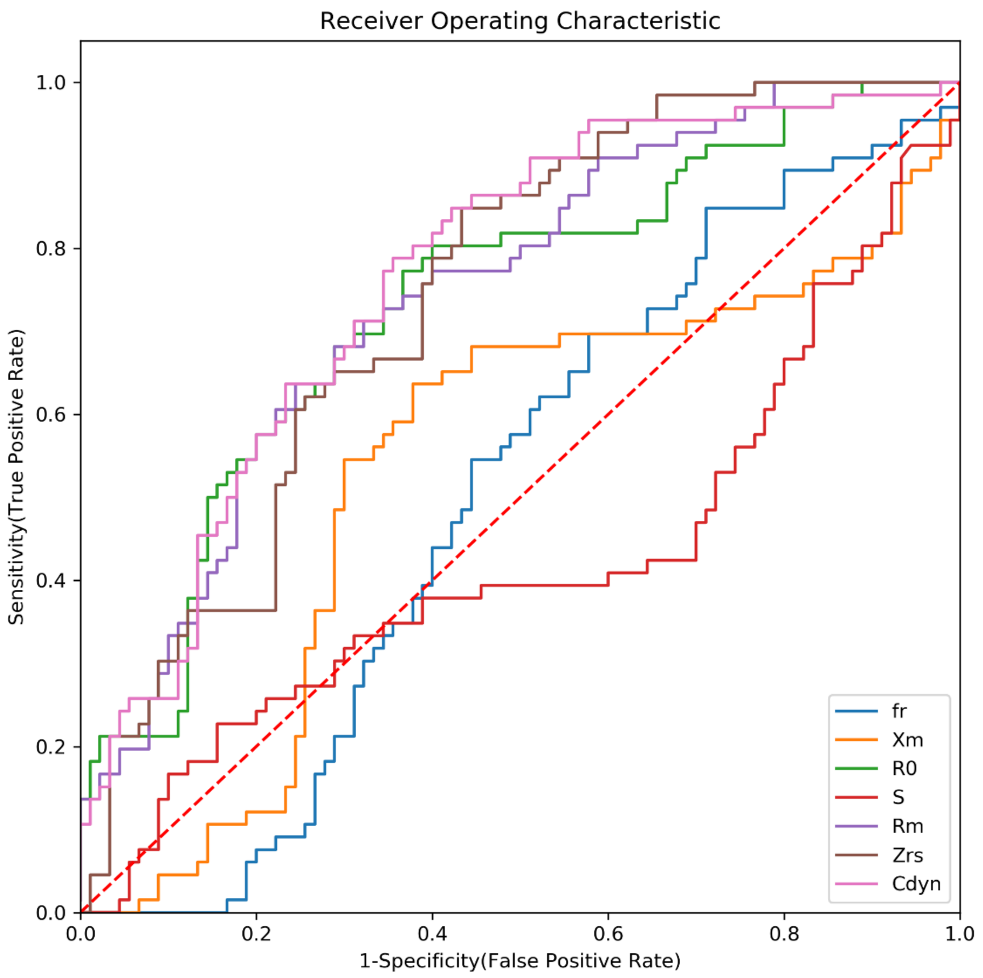

Figure S1: ROC curves for Experiment 1. ROC curves for each one of the FOT parameters - Control Group versus Patients with Sclerosis and Normal Spirometry (CGvsPSNS).

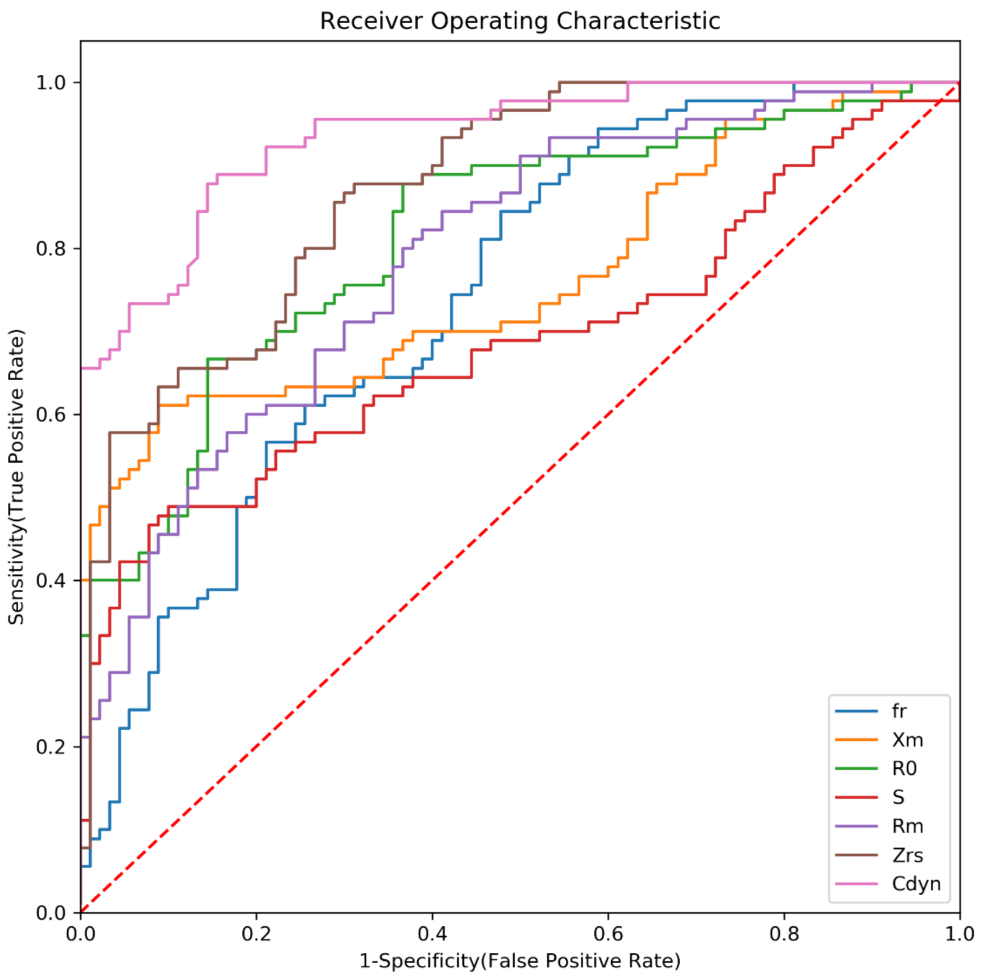

Figure S2: ROC curves for Experiment 1. ROC curves for each one of the FOT parameters - Control Group versus Patients with Sclerosis and Altered Spirometry (CGvsPSAS).

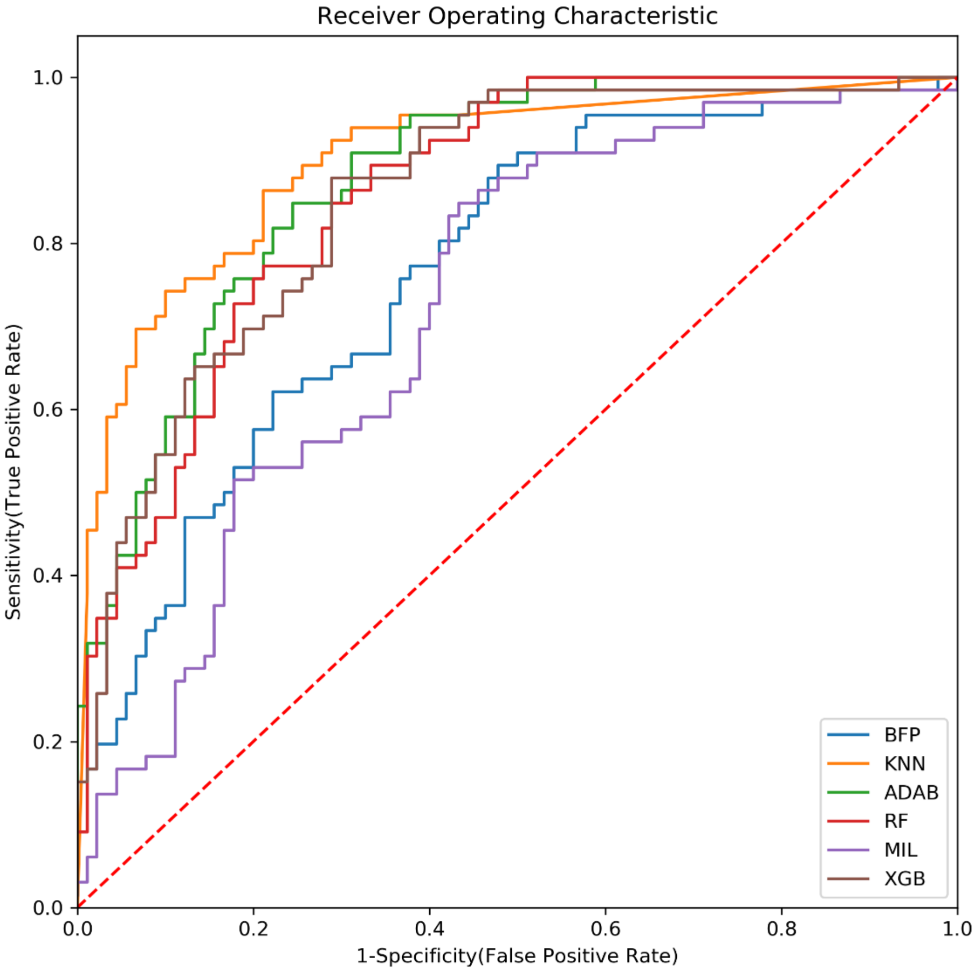

Figure S3: ROC curves for Experiment 2. ROC curves for each one of the FOT parameters - Control Group versus Patients with Sclerosis and Normal Spirometry (CGvsPSNS).

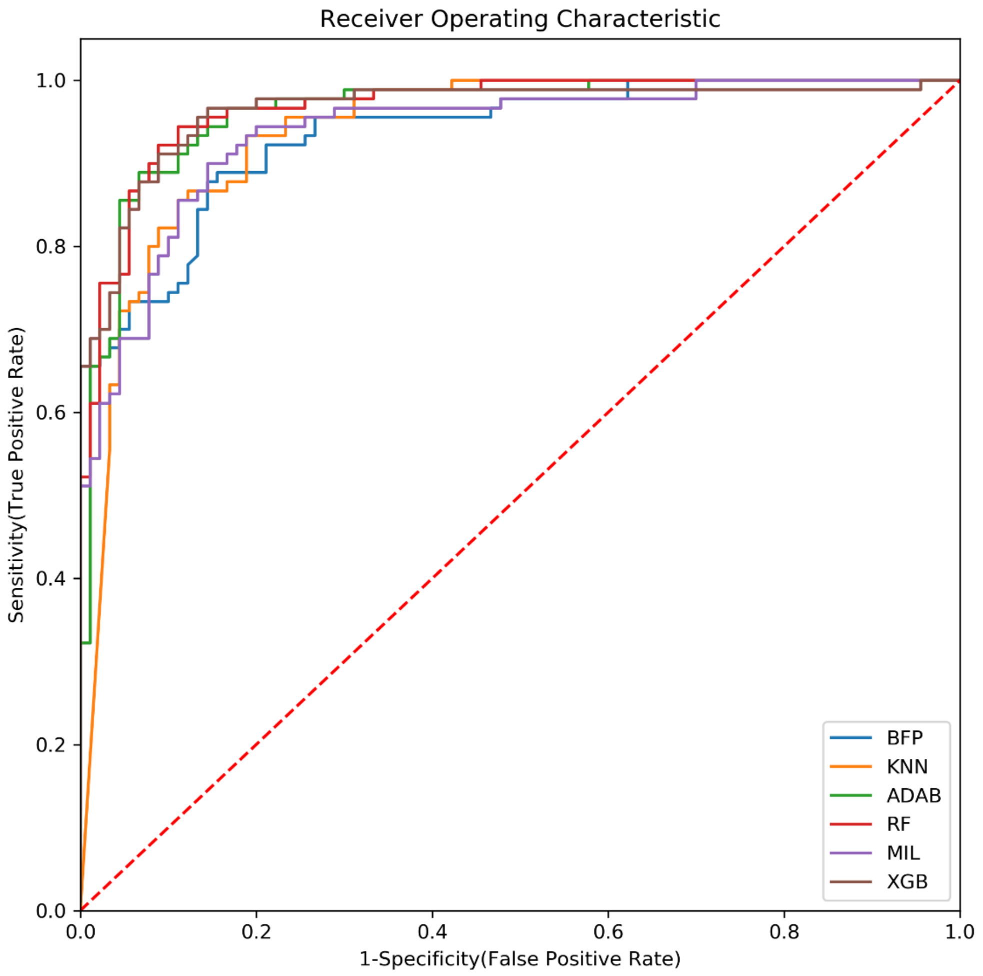

Figure S4: ROC curves for Experiment 2. ROC curves for each one of the FOT parameters - Control Group versus Patients with Sclerosis and Altered Spirometry (CGvsPSAS).

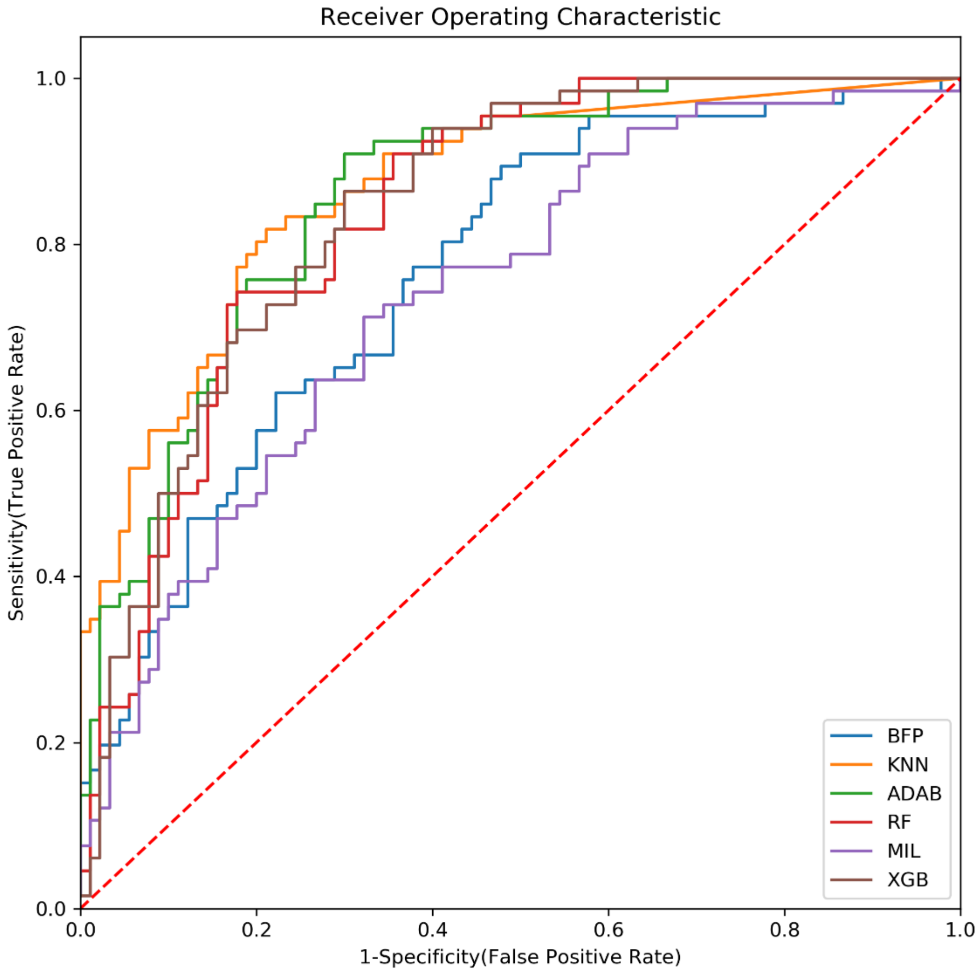

Figure S5: ROC curves for Experiment 3. ROC curves for each one of the FOT parameters - Control Group versus Patients with Sclerosis and Normal Spirometry (CGvsPSNS).

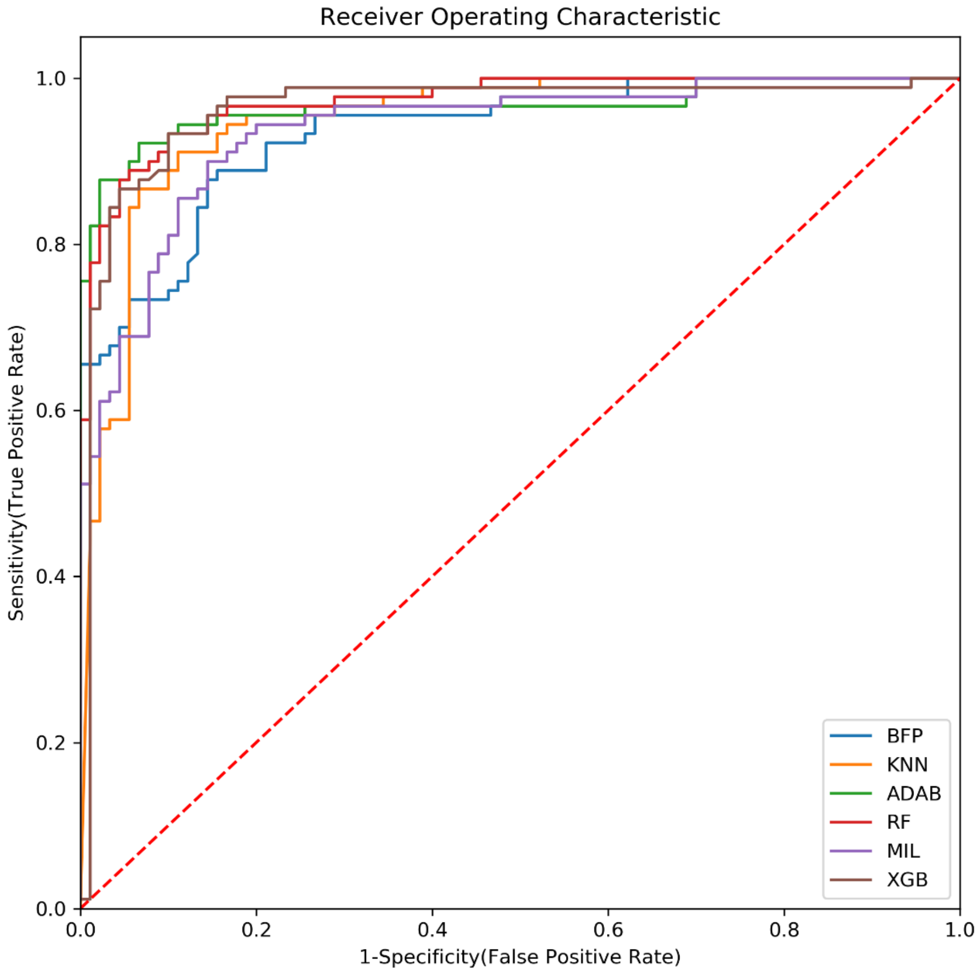

Figure S6: ROC curves for Experiment 3. ROC curves for each one of the FOT parameters - Control Group versus Patients with Sclerosis and Altered Spirometry (CGvsPSAS).

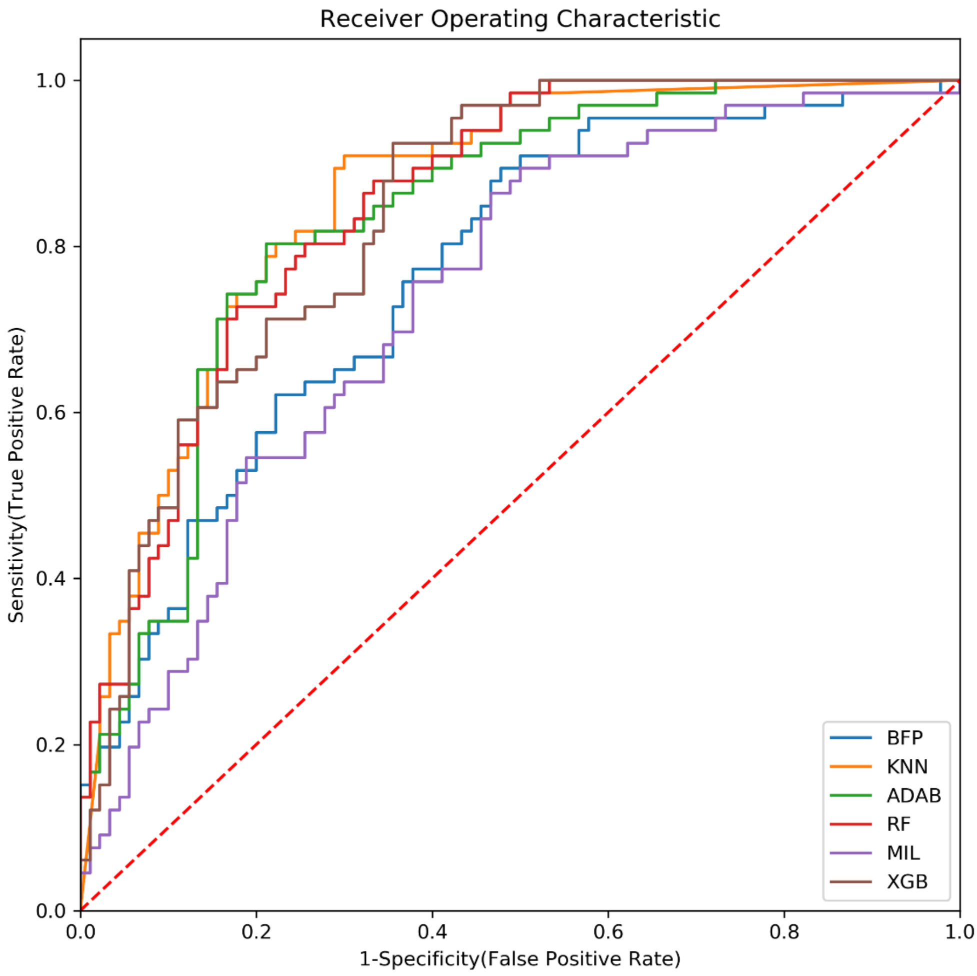

Figure S7: ROC curves for Experiment 4. ROC curves for each one of the FOT parameters - Control Group versus Patients with Sclerosis and Normal Spirometry (CGvsPSNS).

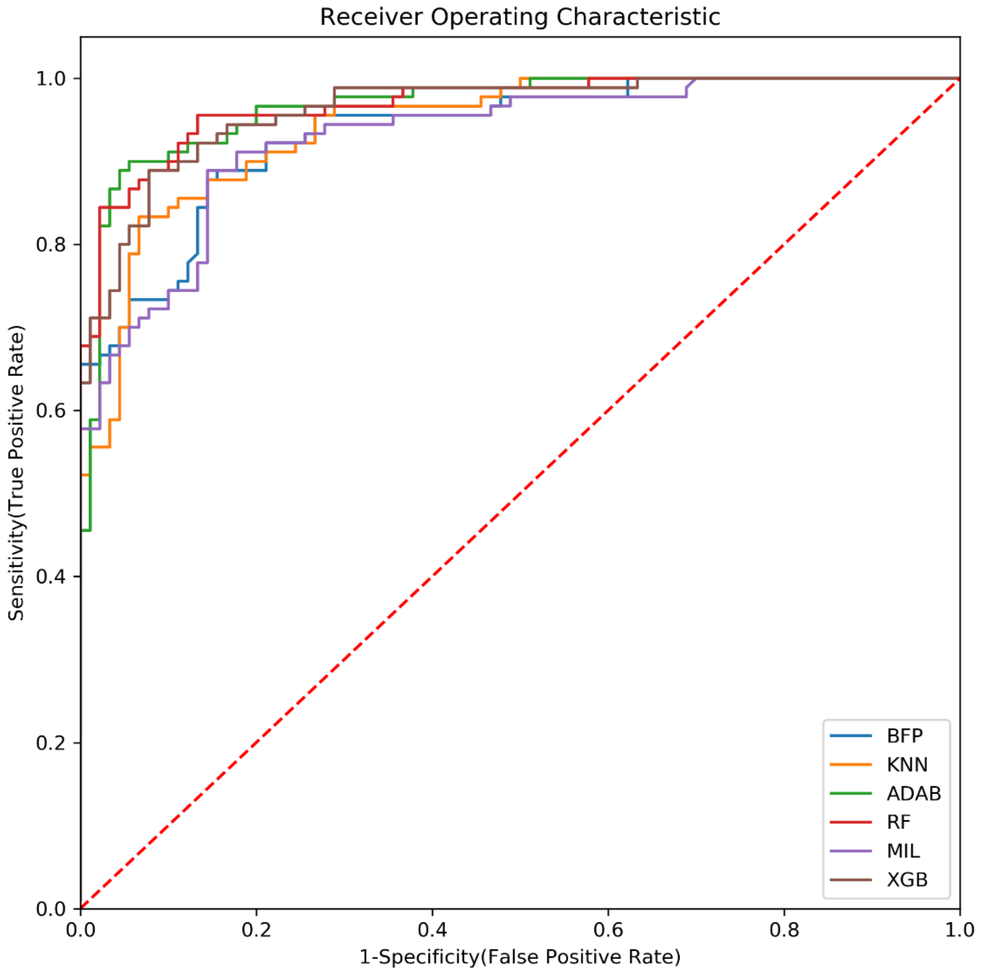

Figure S8: ROC curves for Experiment 4. ROC curves for each one of the FOT parameters - Control Group versus Patients with Sclerosis and Altered Spirometry (CGvsPSAS).

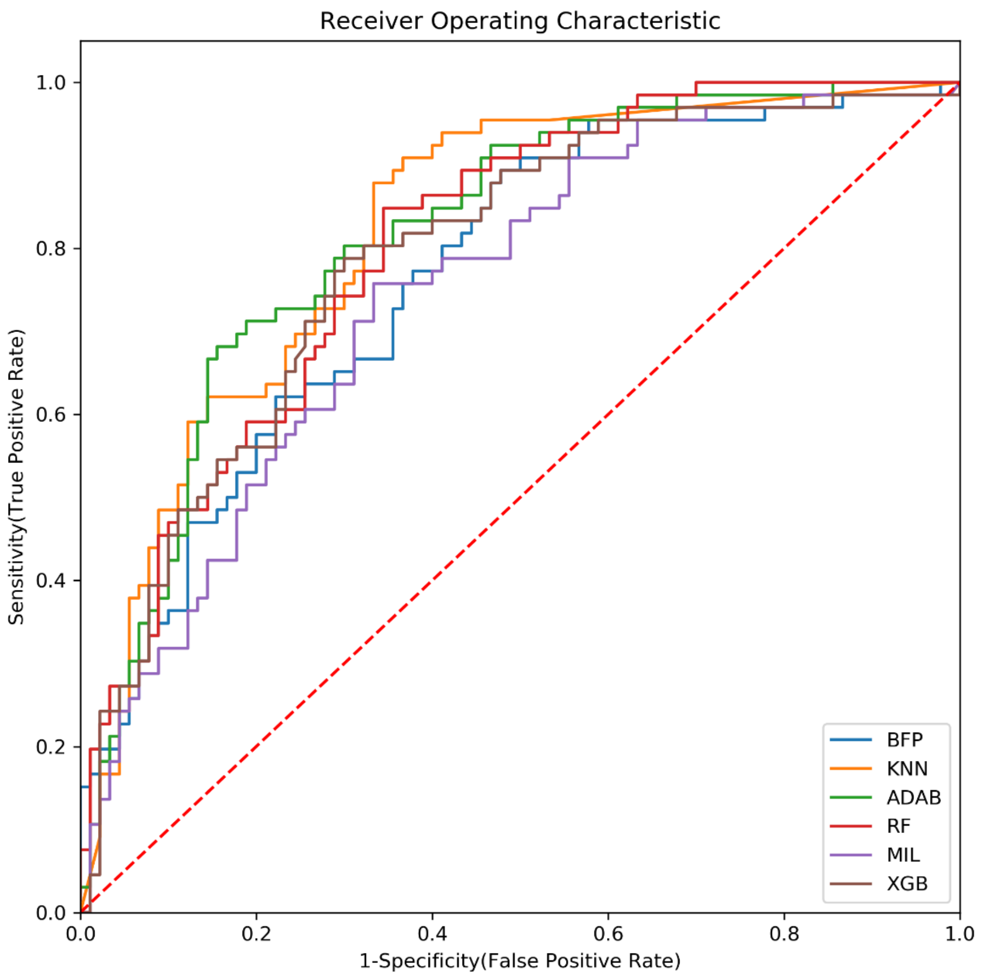

Figure S9: ROC curves for Experiment 5. ROC curves for each one of the FOT parameters - Control Group versus Patients with Sclerosis and Normal Spirometry (CGvsPSNS).

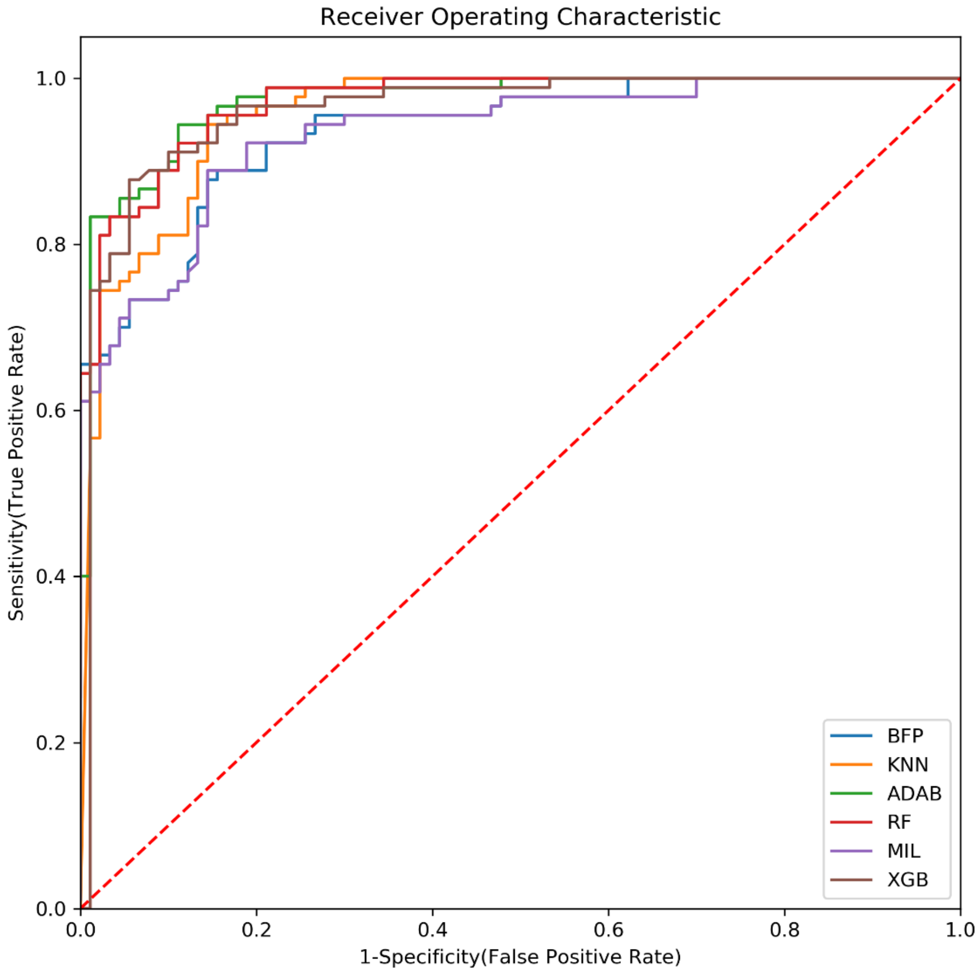

Figure S10: ROC curves for Experiment 5. ROC curves for each one of the FOT parameters - Control Group versus Patients with Sclerosis and Altered Spirometry (CGvsPSAS).

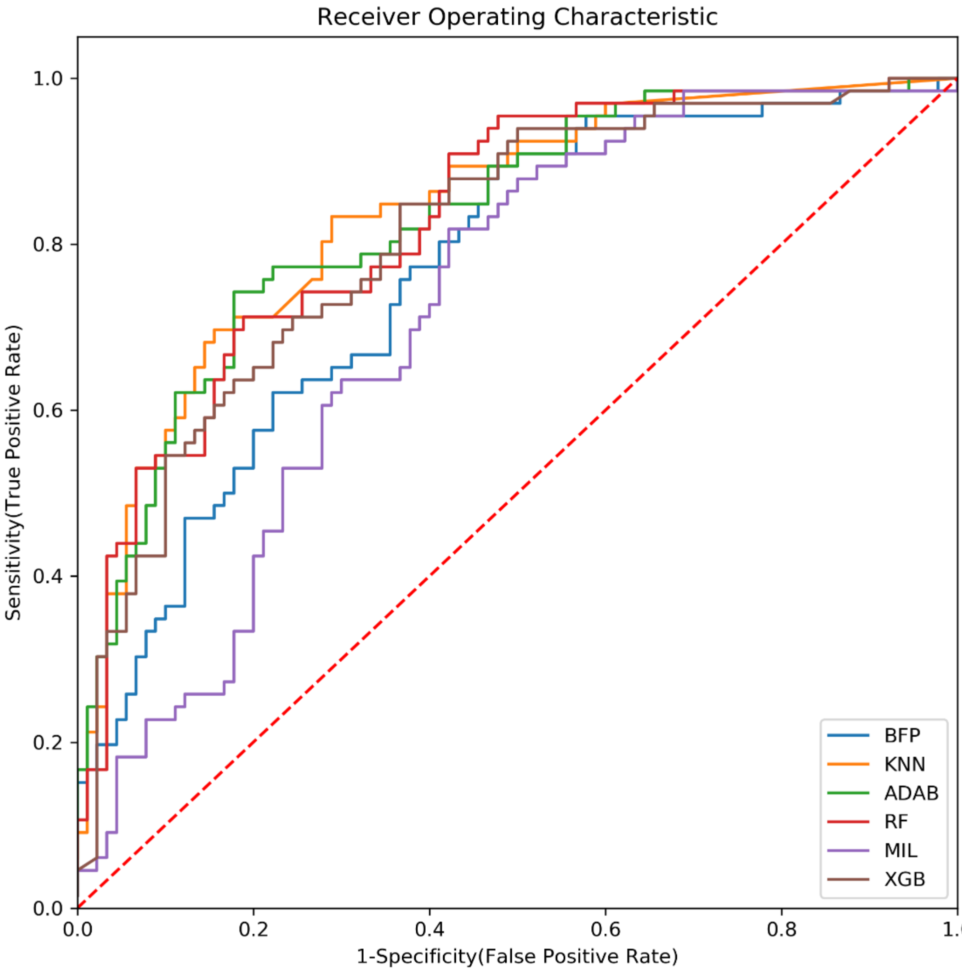

Figure S11: ROC curves for Experiment 6. ROC curves for each one of the FOT parameters - Control Group versus Patients with Sclerosis and Normal Spirometry (CGvsPSNS).

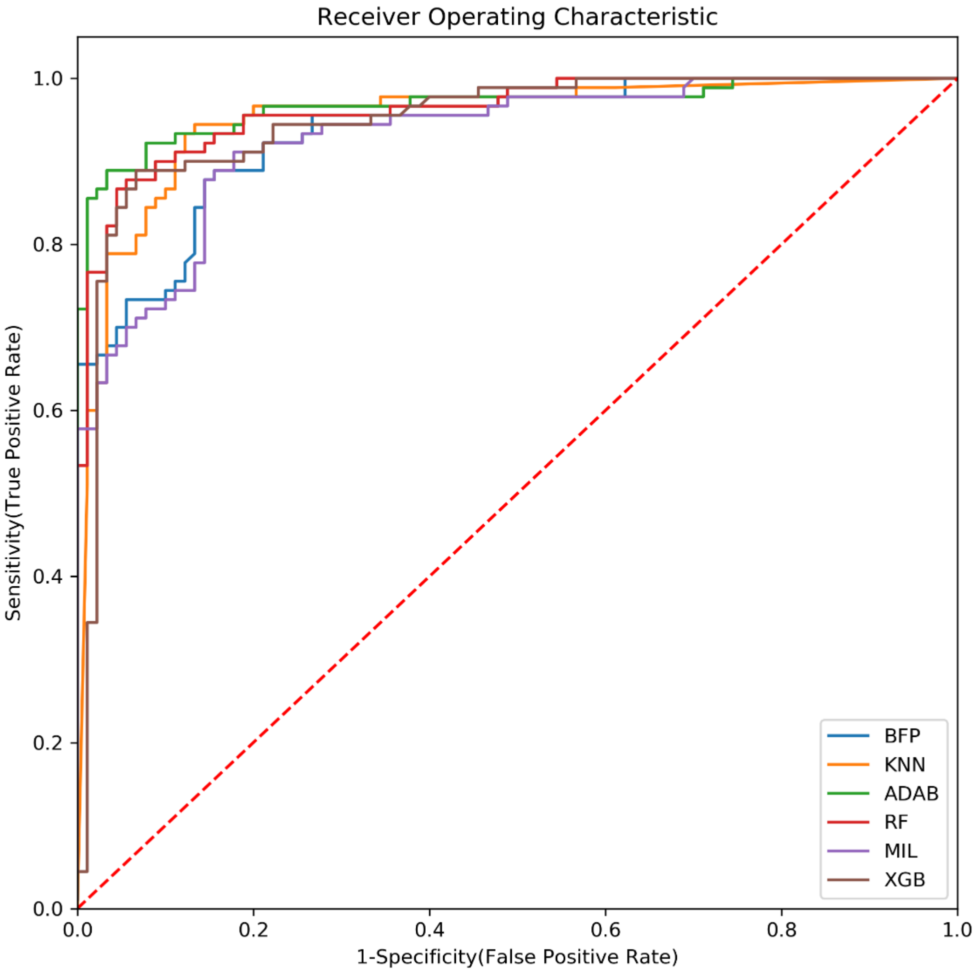

Figure S12: ROC curves for Experiment 6. ROC curves for each one of the FOT parameters - Control Group versus Patients with Sclerosis and Altered Spirometry (CGvsPSAS).

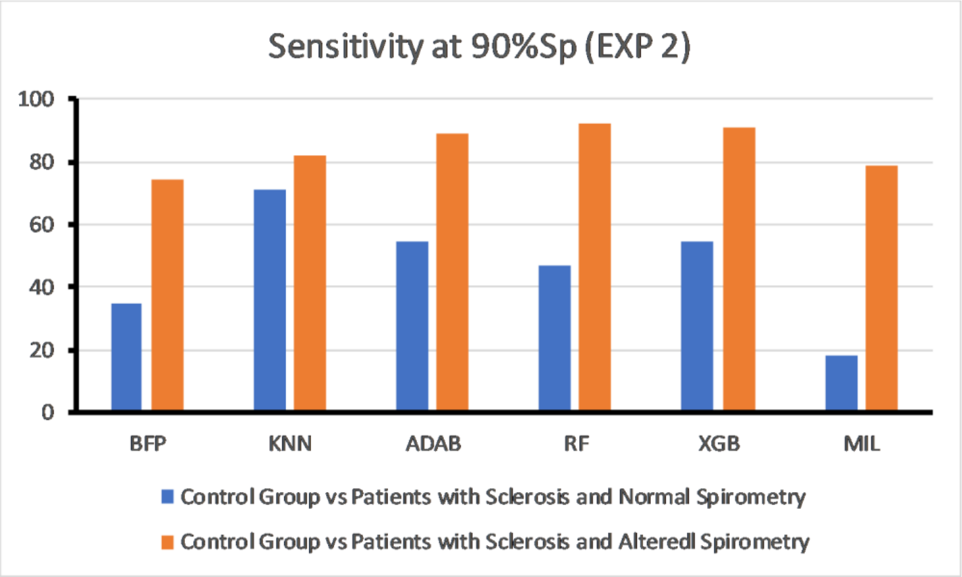

Figure S13: Sensitivity at 90% Sp for Experiment 2.

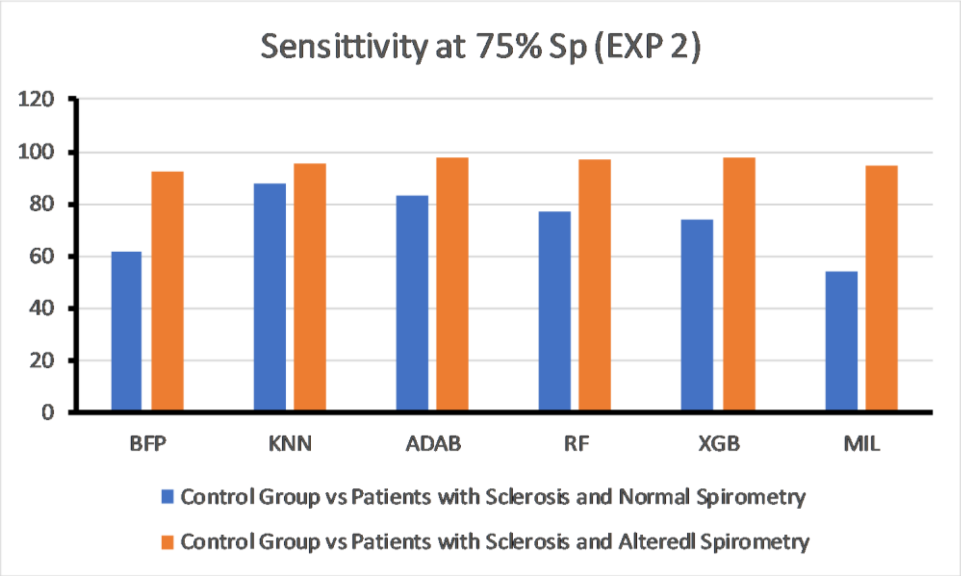

Figure S14: Sensitivity at 75% Sp for Experiment 2.

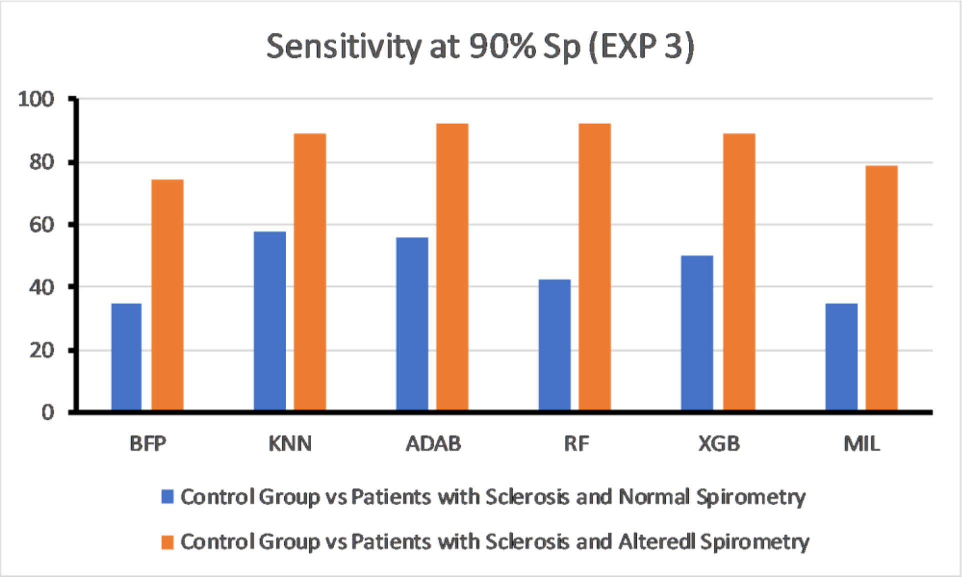

Figure S15: Sensitivity at 90% Sp for Experiment 3.

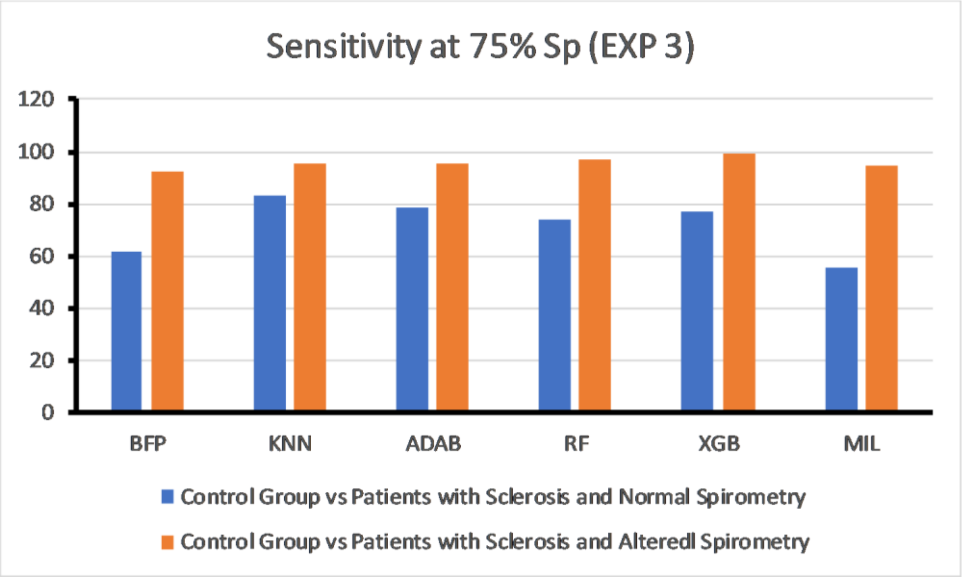

Figure S16: Sensitivity at 75% Sp for Experiment 3.

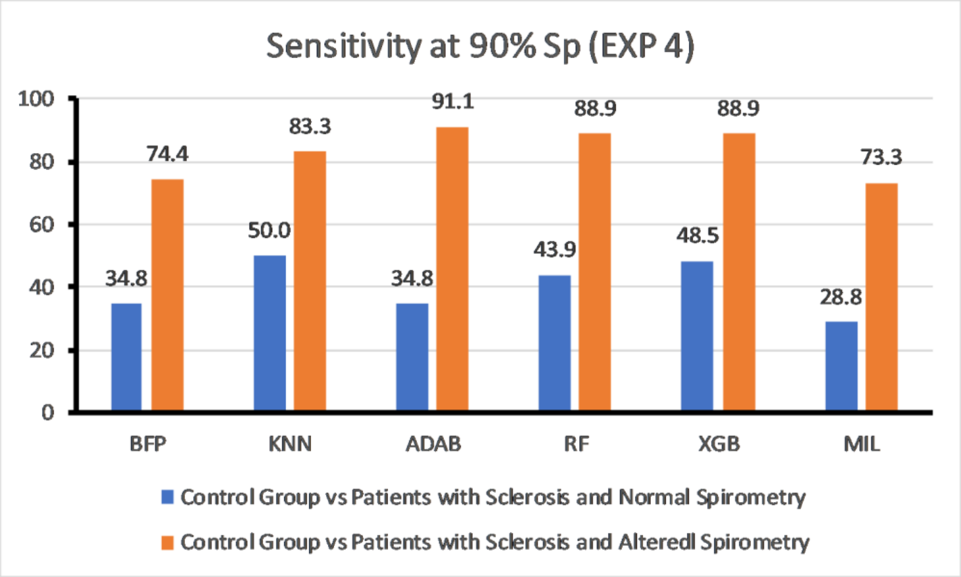

Figure S17: Sensitivity at 90% Sp for Experiment 4.

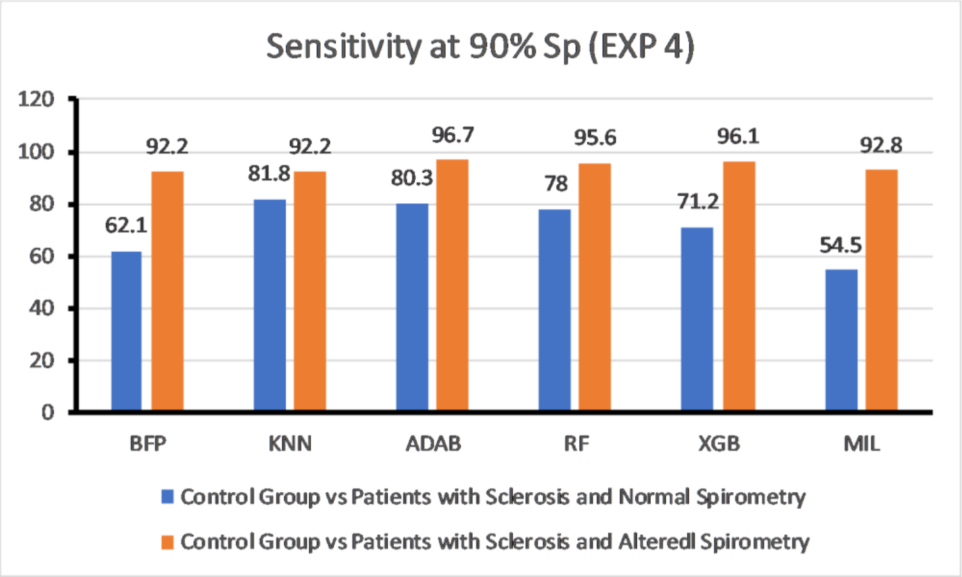

Figure S18: Sensitivity at 75% Sp for Experiment 4.

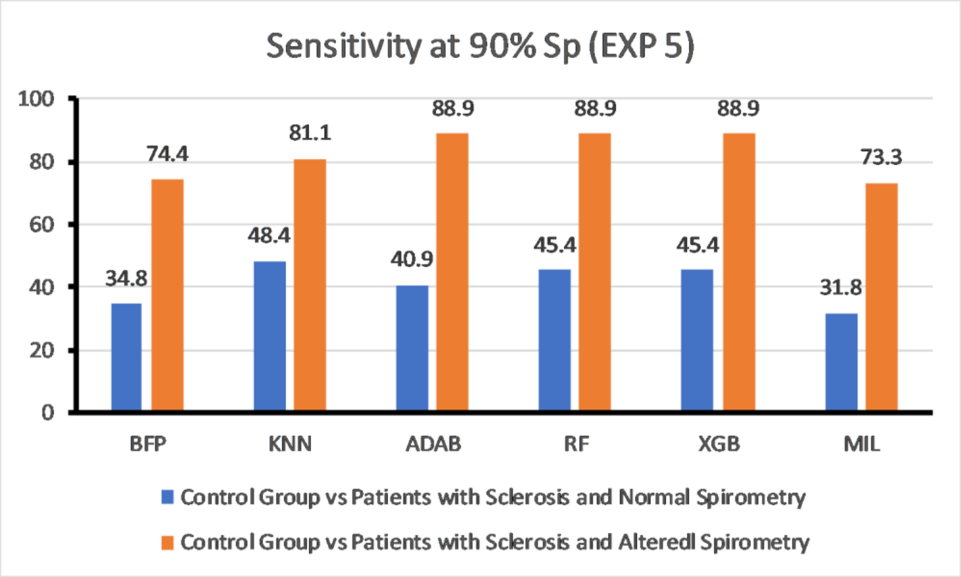

Figure S19: Sensitivity at 90% Sp for Experiment 5.

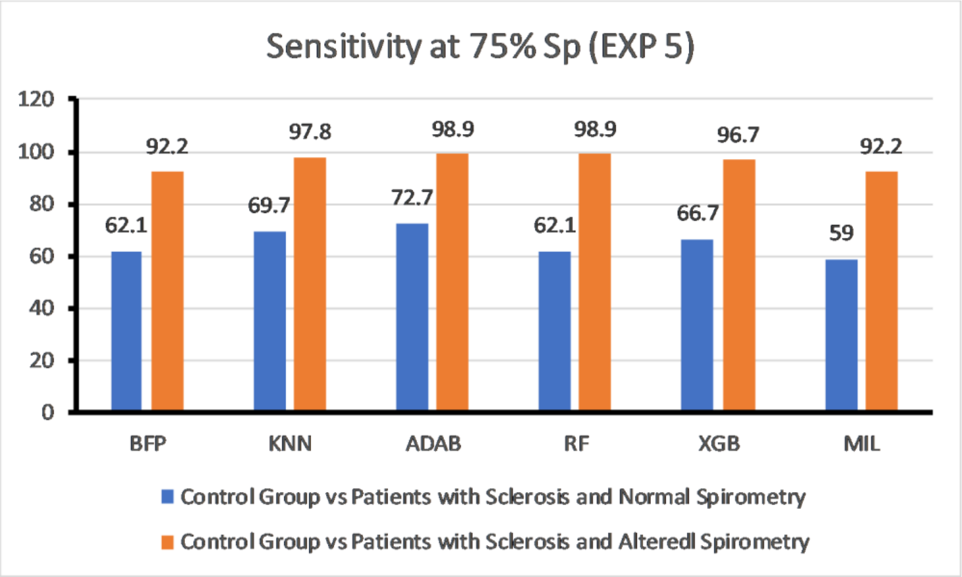

Figure S20: Sensitivity at 75% Sp for Experiment 5.

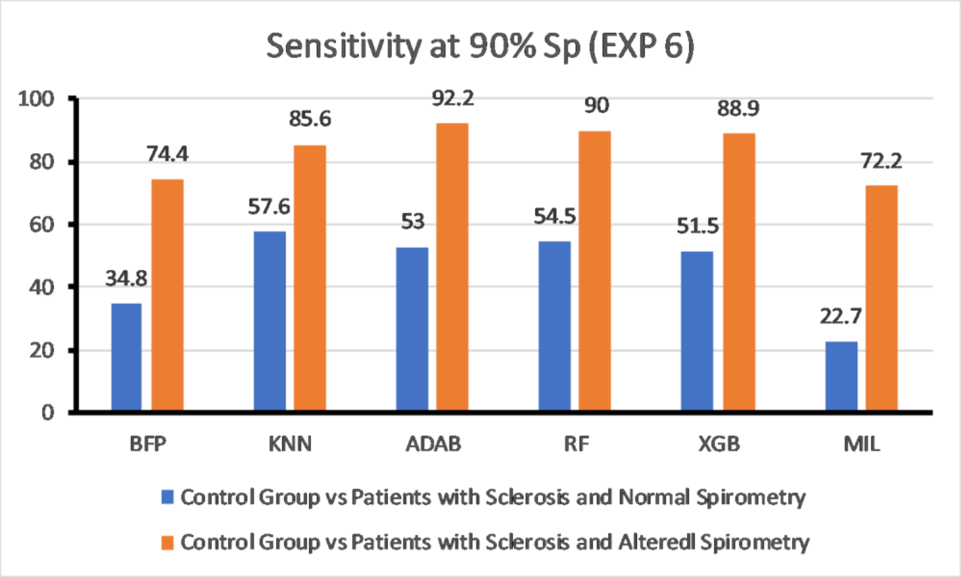

Figure S21: Sensitivity at 90% Sp for Experiment 6.

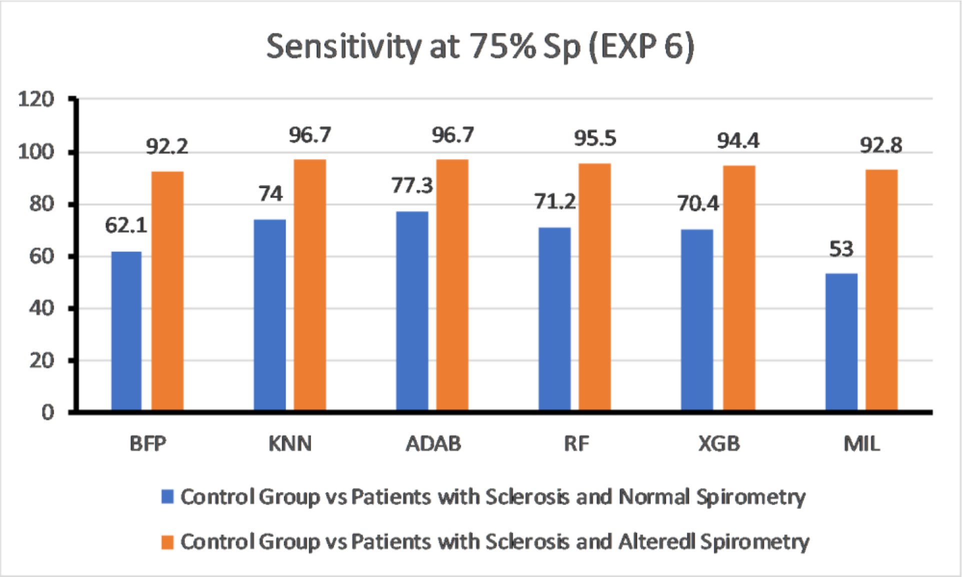

Figure S22: Sensitivity at 75% Sp for Experiment 6.
